# PHLPP isoforms differentially regulate Akt isoforms and AS160 affecting neuronal insulin signaling and insulin resistance via Scribble

**DOI:** 10.1186/s12964-022-00987-0

**Published:** 2022-11-14

**Authors:** Medha Sharma, Chinmoy Sankar Dey

**Affiliations:** Kusuma School of Biological Sciences, Indian Institute of Technology-Delhi, Hauz Khas, New Delhi, 110016 India

**Keywords:** PHLPP1, PHLPP2, Akt1, Akt2, Akt3, AS160, Glucose uptake, Scribble, Neuronal insulin signaling, Neuronal insulin resistance

## Abstract

**Background:**

The aim of the present study was to determine the role of individual PHLPP isoforms in insulin signaling and insulin resistance in neuronal cells.

**Methods:**

PHLPP isoforms were either silenced or overexpressed individually, and the effects were observed on individual Akt isoforms, AS160 and on neuronal glucose uptake, under insulin sensitive and resistant conditions. To determine PHLPP regulation itself, we tested effect of scaffold protein, Scribble, on PHLPP isoforms and neuronal glucose uptake.

**Results:**

We observed elevated expression of both PHLPP1 and PHLPP2 in insulin resistant neuronal cells (Neuro-2A, mouse neuroblastoma; SHSY-5Y, human neuroblastoma) as well as in the whole brain lysates of high-fat-diet mediated diabetic mice. In insulin sensitive condition, PHLPP isoforms differentially affected activation of all Akt isoforms, wherein PHLPP1 regulated serine phosphorylation of Akt2 and Akt3, while PHLPP2 regulated Akt1 and Akt3. This PHLPP mediated Akt isoform specific regulation activated AS160 affecting glucose uptake. Under insulin resistant condition, a similar trend of results were observed in Akt isoforms, AS160 and glucose uptake. Over-expressed PHLPP isoforms combined with elevated endogenous expression under insulin resistant condition drastically affected downstream signaling, reducing neuronal glucose uptake. No compensation was observed amongst PHLPP isoforms under all conditions tested, indicating independent roles and pointing towards possible scaffolding interactions behind isoform specificity. Silencing of Scribble, a scaffolding protein known to interact with PHLPP, affected cellular localization of both PHLPP1 and PHLPP2, and caused increase in glucose uptake.

**Conclusions:**

PHLPP isoforms play independent roles via Scribble in regulating Akt isoforms differentially, affecting AS160 and neuronal glucose uptake.

**Video abstract**

**Supplementary Information:**

The online version contains supplementary material available at 10.1186/s12964-022-00987-0.

## Introduction

Recently we have reported the differential role of Akt isoforms in regulating neuronal insulin signaling and insulin resistance [1]. All Akt isoforms regulated AS160, glucose uptake, and neuronal insulin resistance. However, the relative contribution of each Akt isoform was differential, with Akt2 contributing the most, followed by Akt3 and Akt1. Although Akt2 played the predominant role, we observed a compensatory yet novel role by Akt1 and Akt3 in regulating neuronal insulin signaling and resistance. PHLPP (PH domain Leucine-rich repeat Protein Phosphatase) has previously been studied as primary regulators of Akt isoforms. However, there are paradoxical data regarding PHLPP isoform specific regulation of Akt isoforms. Being a critical upstream phosphatase, the role of PHLPP in neuronal insulin signaling and insulin resistance is largely missing. Thus, role of individual PHLPP isoforms, their regulation of Akt isoforms and possibly other downstream processes is an imminent question.

Brognard et al. reported, for the first time, that PHLPP1 dephosphorylates Akt2 and Akt3, while PHLPP2 targets Akt1 and Akt3 in H157 cell line [2]. Subsequently, similar findings were reported in 293T and SU86 cell lines by Pie et al. [3]. Nitsche et al. reported that PHLPP1 regulates phosphorylation of Akt2, and PHLPP2 regulates phosphorylation of Akt1 in vitro in PaCa cells and in vivo in the orthotopic mouse PaCa model [4]. High levels of PHLPP1 (but not PHLPP2), with corresponding low phosphorylation levels of Akt2 (but not Akt1) regulates pancreatic cell death and tumor formation [4]. In contrast to these reports, Miyamoto et al. reported that in cardiomyocytes, siRNA mediated silencing of PHLPP1 resulted in increased Akt1 and Akt2 phosphorylation, while PHLPP2 silencing did not affect phosphorylation of any Akt isoform [5]. Moc et al. reported that in PHLPP1 knockout mice heart, activity of Akt1 was upregulated (but not Akt2), with corresponding increase in cardiomyocyte size [6]. Thus, differential Akt isoform specific regulation by PHLPP isoforms is highly complex, and warrants further investigations.

PHLPP isoforms are predominantly studied as negative regulators of Akt with a majority of the studies in cancer. There are some studies on PHLPP associated with metabolic disorders. PHLPP1 single-nucleotide polymorphism (SNP) has been recently associated with Type-2 diabetes (T2D) [7, 8]. In skeletal muscles from obese and T2D patients, PHLPP1 mRNA as well as protein level were reported to be elevated as compared to lean participants [9, 10]. Similarly, PHLPP1 protein levels were elevated in adipose tissue of obese patients as well [10]. However, both the studies negate the involvement of PHLPP2 in insulin resistance associated with obesity or diabetes. Recently, only one study reported higher adipocyte PHLPP2 levels in obese mice as compared to lean mice and a possible inter-organ cross-talk with the liver, indicating a role of PHLPP2 in obesity-related metabolic disorders [11]. In the liver, aged and obese mice also display lower PHLPP2 protein levels, affecting Akt signaling and inducing obesity-induced insulin resistance [12]. In pancreas, protein levels of both PHLPP1 and PHLPP2 are elevated in hyperglycemia induced diabetic β-cells [13]. Thus, although PHLPP isoforms have been implicated in metabolic disorders, however, there are paradox regarding PHLPP isoform specific regulation of Akt isoforms. Therefore, to understand and elucidate PHLPP isoform-specific tissue-dependent roles, further studies to determine its possible metabolic implications are warranted.

Although PHLPP1 and PHLPP2 display ubiquitous expression patterns across insulin sensitive tissues, both isoforms are abundantly expressed in the brain as well. Previously, the role of PHLPP has been studied in hippocampal neurons, cortical neurons, etc. in regulating aspects like circadian rhythm [14], neuroprotection [15], brain injury [16, 17], etc. However, there is no report studying the role of PHLPP isoforms in regulating neuronal insulin signaling and insulin resistance. Keeping in mind the above-mentioned tissue-specific complexity, significant role in whole body metabolism under both normal versus pathological conditions, it is imperative to ask PHLPP isoform specific role in neurons, and possible role it might play in terms of insulin signaling and insulin resistance. Since deregulated neuronal insulin signaling leading to neuronal insulin resistance has been linked to many neurodegenerative diseases, the potential role of PHLPP phosphatase is impending.

Another intriguing question is the mechanism behind the regulation of PHLPP isoforms itself. The spatio-temporal association of PHLPP isoform function(s) are studied and scaffolding proteins have been proposed as one of the possible mechanisms of regulation. One such scaffold protein is Scribble [18]. Originally Scribble had been studied as a component of polarity complex (Scrib/Discs large (Dlg)/Lethal giant larvae (Lgl)), and helped maintain proper segregation of membrane proteins and embryonic epithelial polarity in Drosophila [19, 20]. Scribble has also been studied to have a tumor suppressive role [21, 22]. Structurally, Scribble has 16 leucine-rich repeats (LRR) in its amino-terminus and four PDZ domains in its C-terminus, both of which make it an attractive scaffolding target[23]. PHLPP has been reported to form a complex with Scribble, where both PHLPP1 (and to a lesser extent PHLPP2) and Scribble act mutually in regulating phosphorylation of Akt in membrane fractions [18]. Despite reports on PHLPP participation in deregulated metabolic disorders, possible role of Scribble in its association with PHLPP isoforms in such deregulation has not been studied yet.

In our current study we used neuronal cells, insulin sensitive and insulin resistant, like Neuro-2A and SHSY-5Y [24–27] and insulin resistant diabetic mice whole brain lysates to study role of PHLPP in regulating neuronal insulin signaling and insulin resistance. We test this by PHLPP isoform specific silencing as well as over-expression and study its effect on Akt isoforms, AS160 and neuronal glucose uptake under insulin sensitive and insulin resistant condition to elucidate isoform specific functionality. We probed further to study the role of scaffold protein, Scribble, in determining the reason behind the isoform specificity.

## Result

### Effect of insulin stimulation on expression of PHLPP1 and PHLPP2 in insulin signaling and insulin resistant neuronal cells

**Fig. 1:**
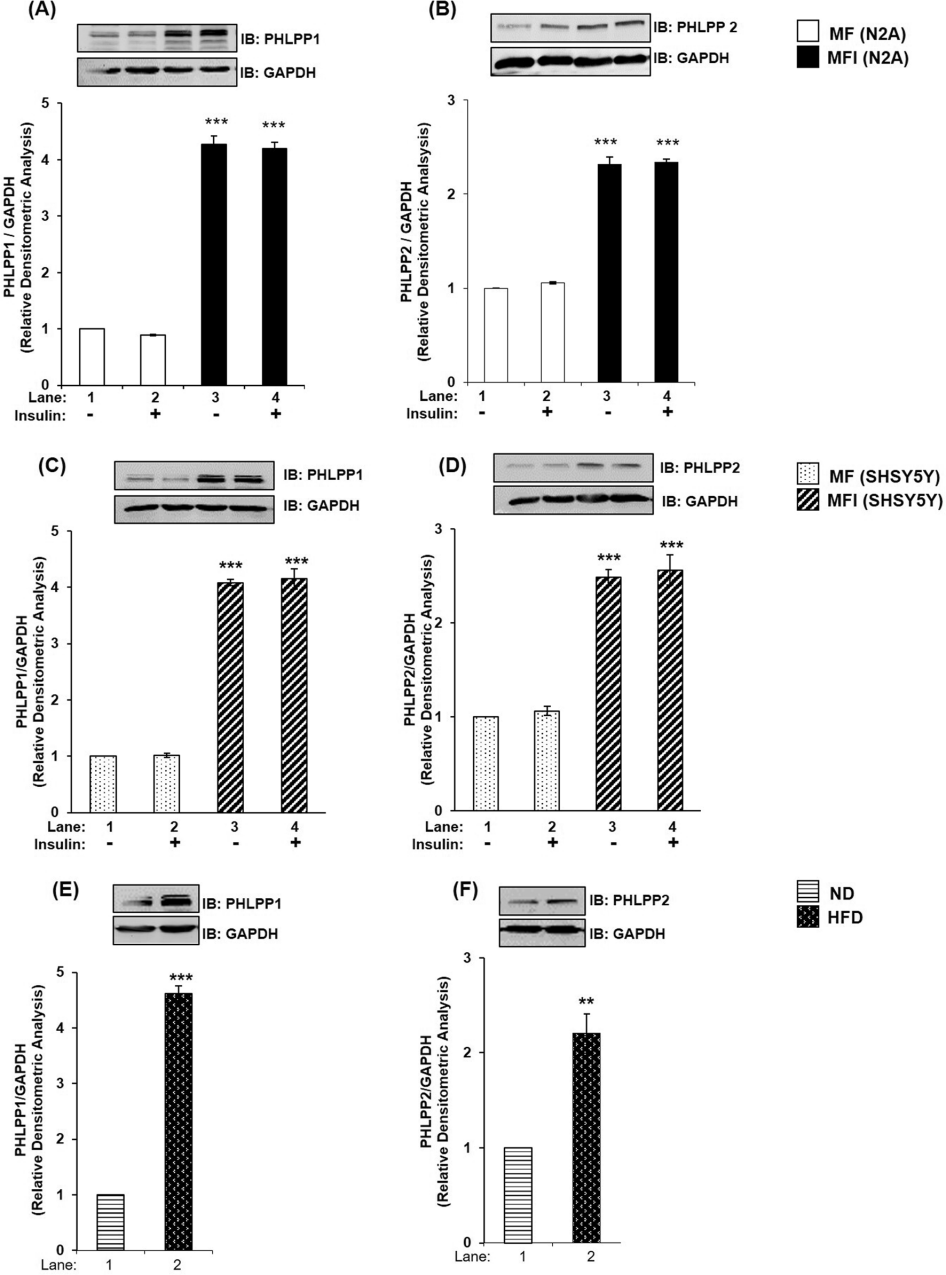
Expression of PHLPP1 and PHLPP2 under insulin resistant condition in neuronal cells. **A–B** N2A cells were differentiated in serum-free medium in the absence of (MF) or chronic presence of 100 nM insulin (MFI) for 3 days. Cells were lysed and subjected to western blotting, followed by probing with relevant primary antibodies. **A** Bar represents relative change in PHLPP1 probed with anti-PHLPP1 antibody. **B** Bar represents relative change in PHLPP2 probed with anti-PHLPP2 antibody. **C–D** SHSY-5Y cells were differentiated in serum-free medium in the absence of (MF) or chronic presence of 100 nM insulin (MFI) for 3 days. Cells were lysed and subjected to western blotting, followed by probing with relevant primary antibodies. **C** Bar represents relative change in PHLPP1 probed with anti-PHLPP1 antibody. **D** Bar represents relative change in PHLPP2 probed with anti-PHLPP2 antibody. **E–F** Mice whole brain was lysed, subjected to western blotting, followed by probing with relevant primary antibodies. **E** Bar represents relative change in PHLPP1 probed with anti-PHLPP1 antibody. **F** Bar represents relative change in PHLPP2 probed with anti-PHLPP2 antibody. GAPDH has been used as a loading control. Experiments were executed three times and a representative result is shown. Data expressed are mean ± SE. ***P < 0.001, **P < 0.01 compared to lane 1 *IB* Immunoblot

We had previously generated an insulin resistant diabetic neuronal cell model by differentiating N2A cells in the chronic presence of insulin (100 nM) in serum-free medium (MFI) [1, 25, 28–30]. In the present study we investigate the expression of PHLPP1 and PHLPP2 in insulin sensitive (MF) and insulin resistant (MFI) neuronal cells. To execute that, insulin sensitive (MF) and insulin resistant (MFI) differentiated N2A cells were stimulated with or without 100 nM insulin for 30 min. Cell lysates were western immunoblotted and probed with either anti-PHLPP1 or anti-PHLPP2 antibody. Under MF condition, insulin stimulation did not affect expression of PHLPP1 (Fig. 1A, lane 1 vs. lane 2; lane 3 vs. lane 4) or PHLPP2 (Fig. 1B, lane 1 vs. lane 2; lane 3 vs. lane 4). However, insulin resistance upregulated expression of PHLPP1 by 4.2 fold (Fig. 1A, lane 1 vs. lane 3) and of PHLPP2 by 2.3 fold (Fig. 1B, lane 1 vs. lane 3) irrespective of insulin stimulation.

To determine whether the above-mentioned effects were cell or species specific, we tested the same in SHSY-5Y cells, a human neuronal cell line. Insulin sensitive (MF) and insulin resistant (MFI) differentiated SHSY-5Y cells displayed results similar to N2A cells (Fig. 1C, D). Expression of both PHLPP1 and PHLPP2 was upregulated under resistant (MFI) condition, however, PHLPP1 expression was more than PHLPP2 in N2A and SHSY-5Y.

### Expression of PHLPP1 and PHLPP2 in High Fat Diet (HFD) fed diabetic mice whole brain tissue lysate

We next tested expression of PHLPP isoforms in whole brain tissue lysate of High-Fat-Diet (HFD) fed diabetic mice. Insulin resistance was generated in sixteen weeks old high-fat-diet (HFD) fed Swiss Albino male mice over ten weeks, and whole brain tissues of those mice, were used for our experiments, as previously reported [1]. Glucose tolerance test and insulin tolerance tests were performed; however, perfusion was not done before brain collection. Mice were divided into two groups: normal diet (ND) and high-fat-diet (HFD), each group containing three animals. Levels of triglyceride, cholesterol, serum glutamic-oxaloacetic transaminase (SGOT), serum glutamic-pyruvic transaminase (SPGT), and fasting blood glucose were elevated by 48%, 29%, 50%, 53%, 40%, respectively in HFD mice as compared to ND mice (kind gifts and information from Dr. Mandal, Indian Institute of Technology-Mandi). In the current study our data showed that, in HFD mice whole brain tissue, expression of PHLPP1 was elevated by 4.5 fold (Fig. 1E, lane 1 vs. lane 2) and of PHLPP2 by 2.2 fold (Fig. 1F, lane 1 vs. lane 2), as compared to respective ND mice whole brain tissue, as observed in resistant neuronal cells.

### Effect of PHLPP1 and PHLPP2 silencing on Akt isoforms and AS160 in insulin signaling and insulin resistant neuronal cells

**Fig. 2:**
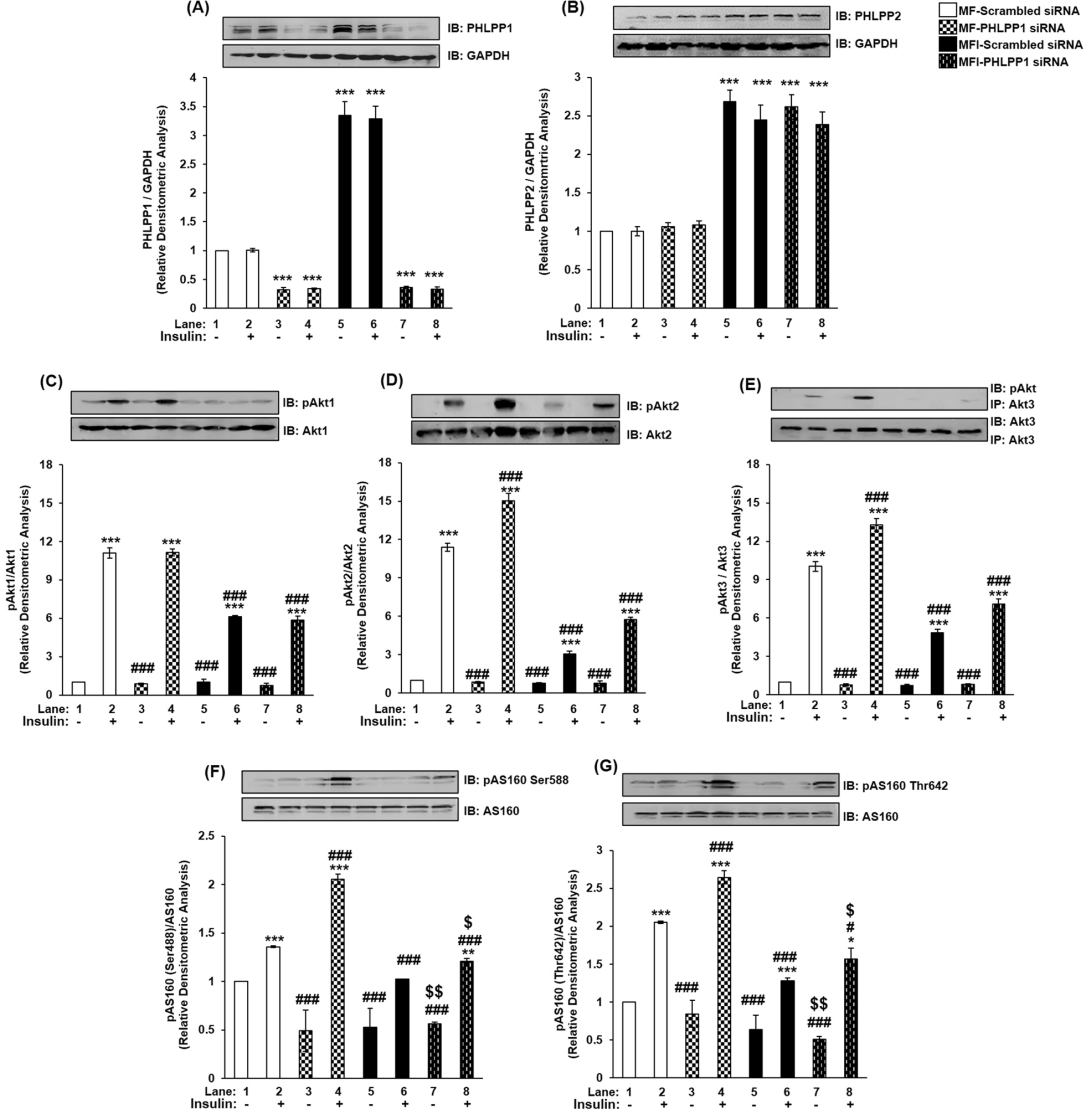
Effect of PHLPP1 silencing on PHLPP isoforms, Akt isoforms and AS160 in insulin signaling and insulin resistant condition in neuronal cells (N2A). Three days post-proliferation, PHLPP1 was silenced using PHLPP1 specific siRNA. N2A cells were differentiated in serum-free medium in the absence of (MF) or chronic presence of 100 nM insulin (MFI) for 3 days. Cells were lysed and subjected to western blotting, followed by probing with relevant primary antibodies. **A** Bar represents relative change in PHLPP1 probed with anti-PHLPP1 antibody. **B** Bar represents relative change in PHLPP2 probed with anti-PHLPP2 antibody. **C** Bar represents relative change in pAkt1 (Ser-473) probed with anti-Akt1 antibody. **D** Bar represents relative change in pAkt2 (Ser-474) probed with anti-Akt2 antibody. **E** Post-insulin stimulation, lysates were subjected to immunoprecipitation using anti-Akt3 antibody. Bar represents relative change in pAkt (Ser-473) probed with anti-Akt3 antibody. **F** Bar represents relative change in pAS160 (Ser-588) when probed with anti-AS160 antibody. **G** Bar represents relative change in pAS160 (Thr-642) when probed with anti-AS160 antibody. GAPDH has been used as a loading control. Experiments were executed three times and a representative result is shown. Data expressed are mean ± SE. ***P < 0.001, **P < 0.01, *P < 0.05 compared to lane 1, ^**###**^*P* < 0.001, ^**#**^*P* < 0.05 as compared to lane 2, ^$$^*P* < 0.01, ^$$^*P* < 0.05 compared to lane 6. *IB* Immunoblot; *IP* Immunoprecipitation

To test the effect of silencing of individual isoforms on insulin signaling, PHLPP1 and PHLPP2 were silenced, and its effect on expression and activation were tested on the downstream substrates, with or without insulin stimulation. Silencing was optimized at 100 nM siRNA for PHLPP1 and PHLPP2 each (Additional files 1, 2: Figs. S1 and S2 respectively). Cells were transfected with 100 nM of either PHLPP1 or PHLPP2 siRNA and then subjected to differentiation under MF or MFI condition, with or without insulin stimulation. Lysates were subjected to western immunoblotting probed with relevant antibodies as and when mentioned with reference to the relevant experiment. PHLPP1 silencing decreased PHLPP1 expression by 70% under MF and MFI conditions (Fig. 2A, lane 1 vs. lane 3, 4; lane 1 vs. lane 7, 8) as compared to scrambled siRNA transfected cells. Consistent with our previous result (Fig. 1A, lane 1 vs. lane 5, 6), expression of PHLPP1 displayed increase under MFI condition, however, silenced PHLPP1 was effective under both conditions tested. PHLPP1 silencing did not affect expression of PHLPP2 under MF or MFI condition (Fig. 2B). Previously, Chen et al. had reported in mice brain, that PHLPP1 knockout did not lead to compensatory increase in PHLPP2 expression [16].

We have previously reported insulin resistance dependent decrease in serine phosphorylation of Akt1, Akt2 and Akt3 in neuronal cells [1].We next proceeded to test the effect of PHLPP1 silencing on Akt isoforms in neuronal insulin signaling and resistance. Post PHLPP1 silencing, there was no change in expression of Akt1 under all the conditions tested (Fig. 2C). Under control conditions (scrambled siRNA transfected) activation of Akt1, as determined by phosphorylation at Ser-473, was down-regulated from MF to MFI by 44% (Fig. 2C, lane 2 vs. lane 6). However, due to PHLPP1 silencing, activation of Akt1 was unaffected under MF and MFI conditions (Fig. 2C, lane 2 vs. lane 4; lane 6 vs. lane 8). Thus, PHLPP1 does not affect phosphorylation or expression of Akt1 in neuronal insulin signaling and resistance. PHLPP1 silencing, caused no change in expression of Akt2 under all the conditions tested (Fig. 2D). However, under all control conditions (scrambled siRNA transfected) activation of Akt2, as determined by phosphorylation at Ser-474, was down-regulated from MF to MFI conditions by 73% (Fig. 2D, lane 2 vs. lane 6). Due to PHLPP1 silencing, activation of Akt2 was up-regulated by insulin stimulation by 32% (Fig. 2D, lane 2 vs. lane 4), and by 87% (Fig. 2D, lane 6 vs. lane 8) under MF and MFI conditions, respectively. Post PHLPP1 silencing, there was no change in expression of Akt3 under all the conditions tested (Fig. 2E). However, under control conditions (scrambled siRNA transfected) activation of Akt3, as determined by phosphorylation at Ser-472, was down-regulated from MF to MFI conditions by 52% (Fig. 2E, lane 2 vs. lane 6). Due to PHLPP1 silencing, activation of Akt3 was up-regulated by insulin stimulation by 32% (Fig. 2E, lane 2 vs. lane 4), and by 46% (Fig. 2E, lane 6 vs. lane 8) under MF and MFI conditions, respectively. Data shows that PHLPP1 specifically regulates phosphorylation of Akt2 and Akt3, without affecting expression in insulin sensitive and resistant conditions. Additionally, PHLPP1 does not regulate phosphorylation or expression of Akt1 in insulin signaling.

We had previously established how Akt isoforms regulate AS160 in neuronal insulin signaling and insulin resistance [1]. All Akt isoforms regulate serine and threonine phosphorylation of AS160, with Akt2 contributing the most, followed by Akt3 and Akt1 under MF and MFI condition [1]. Now, having studied PHLPP1 specific role in regulating Akt isoforms, the next question was whether this regulation extends to AS160. AS160 is a Rab-GAP (GTPase activating protein) involved in glucose transporter 4 (GLUT4) translocation [1]. Post PHLPP1 silencing, there was no change in expression of AS160 under all the conditions tested (Fig. 2F, G). However, under all control conditions (scrambled siRNA transfected) activation of AS160, as determined by phosphorylation at Ser-588 and Thr-642, was down-regulated from MF to MFI conditions by 24% and 37%, respectively (Fig. 2F, G, lane 2 vs. lane 6). Due to PHLPP1 silencing, activation of AS160 at Ser-588 was up-regulated by insulin stimulation by 51% (Fig. 2F, lane 2 vs. lane 4), and by 17% (Fig. 2F, lane 6 vs. lane 8) under MF and MFI conditions, respectively. Similarly, activation of AS160 at Thr-642 was up-regulated by insulin stimulation by 29% (Fig. 2G, lane 2 vs. lane 4), and by 21% (Fig. 2G, lane 6 vs. lane 8) under MF and MFI conditions, respectively. Previously, PHLPP isoforms have been reported to regulate substrate specificity and amplitude of various substrates of Akt isoforms, but AS160 downstream of PHLPP1 has never been studied in any tissue system before. Thus, we report PHLPP1 specific regulation of Akt isoforms which extends to regulation of AS160 serine and threonine phosphorylation, more so, in a neuronal insulin resistant system. 

**Fig. 3:**
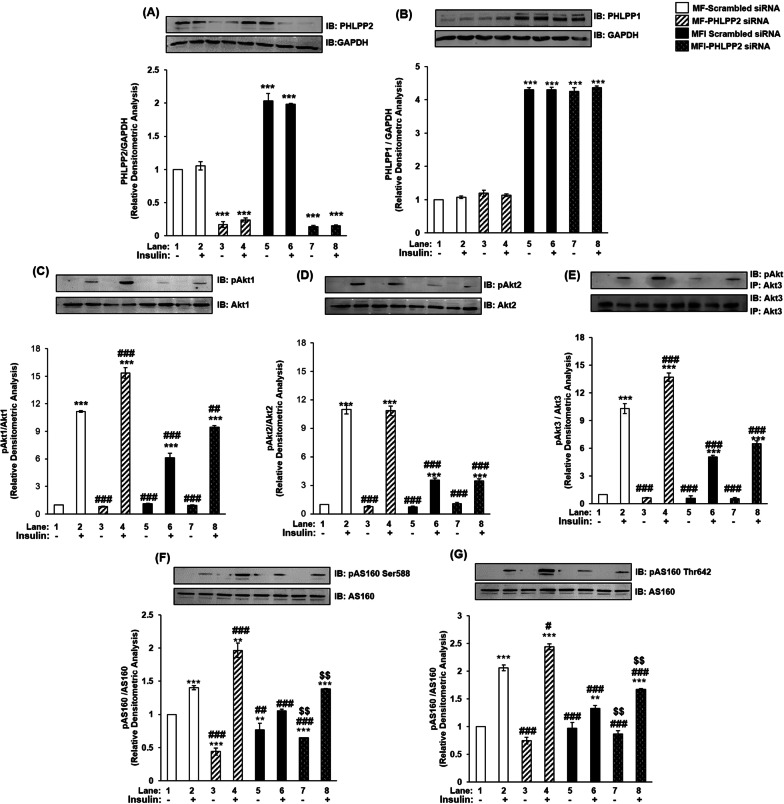
Effect of PHLPP2 silencing on PHLPP isoforms, Akt isoforms and AS160 in insulin signaling and insulin resistant condition in neuronal cells (N2A). Three days post-proliferation, PHLPP2 was silenced using PHLPP2 specific siRNA. N2A cells were differentiated in serum-free medium in the absence of (MF) or chronic presence of 100 nM insulin (MFI) for 3 days. Cells were lysed and subjected to western blotting, followed by probing with relevant primary antibodies. **A** Bar represents relative change in PHLPP2 probed with anti-PHLPP2 antibody. **B** Bar represents relative change in PHLPP1 probed with anti-PHLPP1 antibody. **C** Bar represents relative change in pAkt1 (Ser-473) probed with anti-Akt1 antibody. **D** Bar represents relative change in pAkt2 (Ser-474) probed with anti-Akt2 antibody. **E** Post-insulin stimulation, lysates were subjected to immunoprecipitation using anti-Akt3 antibody. Bar represents relative change in pAkt (Ser-473) probed with anti-Akt3 antibody. **F** Bar represents relative change in pAS160 (Ser-588) when probed with anti-AS160 antibody. **G** Bar represents relative change in pAS160 (Thr-642) when probed with anti-AS160 antibody. GAPDH has been used as a loading control. Experiments were executed three times and a representative result is shown. Data expressed are mean ± SE. ***P < 0.001, **P < 0.01, *P < 0.05 compared to lane 1, ^**###**^*P* < 0.001, ^**##**^*P* < 0.01, ^**#**^*P* < 0.05 as compared to lane 2, ^$$^*P* < 0.01 compared to lane 6 *IB* Immunoblot; *IP* Immunoprecipitation

Having observed, as above, it was imperative to study role of PHLPP2 as well. PHLPP2 silencing decreased PHLPP2 expression by 80% under MF and MFI conditions, respectively (Fig. 3A, lane 1 vs. lane 3, 4; and lane 1 vs. lane 5, 6) as compared to scrambled siRNA transfected cells. Consistent with our previous result (Fig. 1B, lane 1 vs. lane 5, 6), expression of PHLPP2 displayed increase under MFI condition, however, silenced PHLPP2 was effective under both conditions tested. PHLPP2 silencing did not affect expression of PHLPP1 under MF or MFI condition (Fig. 3B). This essentially points to independent roles played by PHLPP isoforms without any compensatory upregulation in expression.

PHLPP2 has also been reported to regulate Akt isoforms specifically in cancer cells [2, 3]. However, unlike PHLPP1, which has been studied as an upstream regulator of Akt isoforms in insulin resistant systems [9, 10], data on PHLPP2 is very limited. Post PHLPP2 silencing, we observed no change in expression of Akt1 under all the conditions tested (Fig. 3C). However, PHLPP2 silencing up-regulated activation of Akt1 post insulin stimulation by 37% (Fig. 3C, lane 2 vs. lane 4), and by 54% (Fig. 3C, lane 6 vs. lane 8) under MF and MFI conditions, respectively. There was no change in expression of Akt2 post PHLPP2 silencing under all the conditions tested (Fig. 3D). Interestingly, due to PHLPP2 silencing, activation of Akt2 was unaffected under MF and MFI conditions (Fig. 3D, lane 2 vs. lane 4; lane 6 vs. lane 8). Post PHLPP2 silencing, there was no change in expression of Akt3 under all the conditions tested (Fig. 3E). Due to PHLPP2 silencing, activation of Akt3 was also up-regulated by insulin stimulation by 33% (Fig. 3E, lane 2 vs. lane 4), and by 51% (Fig. 3E, lane 6 vs. lane 8) under MF and MFI conditions, respectively. Data demonstrates that isoforms of PHLPP regulate isoforms of Akt in insulin sensitive and insulin resistant neuronal insulin signaling.

PHLPP2 silencing caused no change in expression of AS160 under all the conditions tested (Fig. 3F, G). However, under all control conditions (scrambled siRNA transfected) activation of AS160 was down-regulated from MF to MFI conditions by 24% and 35%, respectively (Fig. 3F, G, lane 2 vs. lane 6). Due to PHLPP2 silencing, activation of AS160 at Ser-588 was up-regulated by insulin stimulation by 40% (Fig. 3F, lane 2 vs. lane 4), and by 31% (Fig. 3F, lane 6 vs. lane 8) under MF and MFI conditions, respectively. Similarly, activation of AS160 at Thr-642 was up-regulated by insulin stimulation by 18% (Fig. 3G, lane 2 vs. lane 4), and by 25% (Fig. 3G, lane 6 vs. lane 8) under MF and MFI conditions, respectively. AS160 regulation by PHLPP isoforms has never been studied before. Data on the effects of PHLPP1 and PHLPP2 silencing on downstream substrates has been summarized in Additional file 6: Table S1. Data consolidates that two isoforms of PHLPP regulate three isoforms of Akt in insulin sensitive and insulin resistant neuronal insulin signaling.

### Effect of PHLPP1 and PHLPP2 over-expression on Akt isoforms and AS160 in insulin signaling and insulin resistant neuronal cells

Having seen how PHLPP isoforms regulate Akt isoforms and AS160 by PHLPP isoform specific silencing, we proceeded to test the effect of overexpression of PHLPP isoform as well. We transfected each isoform individually in cells and transfected cells were then subjected to MF and MFI conditions, and the effect of over-expression was tested with or without insulin stimulation. Lysates were subjected to western immunoblotting probed with relevant antibodies as and when mentioned with reference to the relevant experiment.

**Fig. 4:**
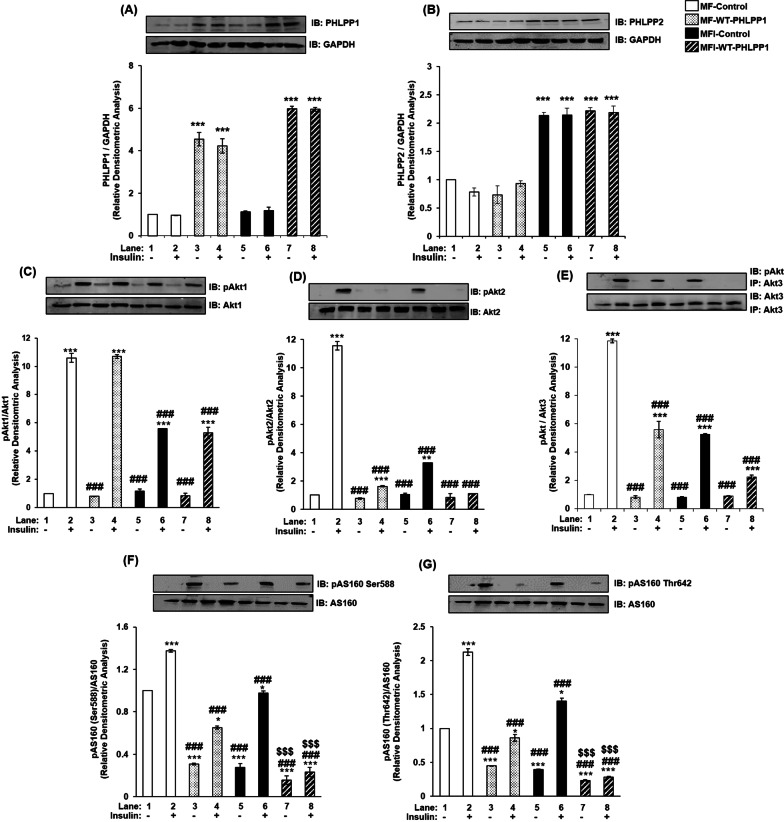
Effect of PHLPP1 over-expression on PHLPP isoforms, Akt isoforms and AS160 in insulin signaling and insulin resistant condition in neuronal cells (N2A). Three days post-proliferation, PHLPP1 was over-expressed using PHLPP1 specific plasmid. N2A cells were differentiated in serum-free medium in the absence of (MF) or chronic presence of 100 nM insulin (MFI) for 3 days. Cells were lysed and subjected to western blotting, followed by probing with relevant primary antibodies. **A** Bar represents relative change in PHLPP1 probed with anti-PHLPP1 antibody. **B** Bar represents relative change in PHLPP2 probed with anti-PHLPP2 antibody. **C** Bar represents relative change in pAkt1 (Ser-473) probed with anti-Akt1 antibody. **D** Bar represents relative change in pAkt2 (Ser-474) probed with anti-Akt2 antibody. **E** Post-insulin stimulation, lysates were subjected to immunoprecipitation using anti-Akt3 antibody. Bar represents relative change in pAkt (Ser-473) probed with anti-Akt3 antibody. **F** Bar represents relative change in pAS160 (Ser-588) when probed with anti-AS160 antibody. **G** Bar represents relative change in pAS160 (Thr-642) when probed with anti-AS160 antibody. GAPDH has been used as a loading control. Experiments were executed three times and a representative result is shown. Data expressed are mean ± SE. ***P < 0.001, **P < 0.01, *P < 0.05 compared to lane 1, ^**###**^*P* < 0.001, ^**##**^*P* < 0.01, ^**#**^*P* < 0.05 as compared to lane 2, ^$$$^*P* < 0.001 as compared to lane 6. *IB* Immunoblot; *IP* Immunoprecipitation

**Fig. 5:**
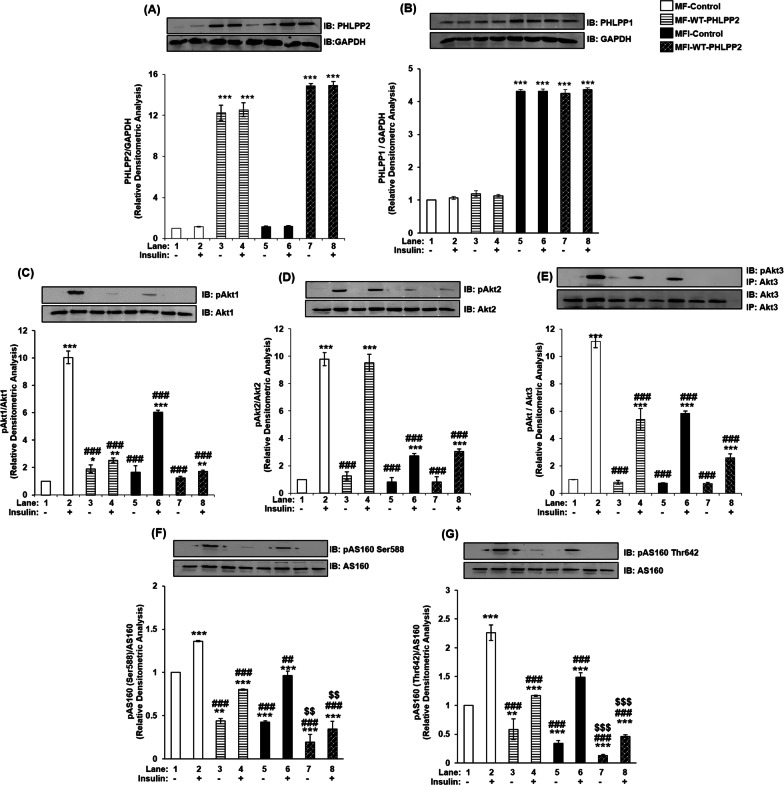
Effect of PHLPP2 over-expression on PHLPP isoforms, Akt isoforms and AS160 in insulin signaling and insulin resistant condition in neuronal cells (N2A). Three days post-proliferation, PHLPP2 was over-expressed using PHLPP2 specific plasmid. N2A cells were differentiated in serum-free medium in the absence of (MF) or chronic presence of 100 nM insulin (MFI) for 3 days. Cells were lysed and subjected to western blotting, followed by probing with relevant primary antibodies. **A** Bar represents relative change in PHLPP2 probed with anti-PHLPP2 antibody. **B** Bar represents relative change in PHLPP1 probed with anti-PHLPP1 antibody. **C** Bar represents relative change in pAkt1 (Ser-473) probed with anti-Akt1 antibody. **D** Bar represents relative change in pAkt2 (Ser-474) probed with anti-Akt2 antibody. **E** Post-insulin stimulation, lysates were subjected to immunoprecipitation using anti-Akt3 antibody. Bar represents relative change in pAkt (Ser-473) probed with anti-Akt3 antibody. **F** Bar represents relative change in pAS160 (Ser-588) when probed with anti-AS160 antibody. **G** Bar represents relative change in pAS160 (Thr-642) when probed with anti-AS160 antibody. GAPDH has been used as a loading control. Experiments were executed three times and a representative result is shown. Data expressed are mean ± SE. ***P < 0.001, **P < 0.01, *P < 0.05 compared to lane 1, ^###^*P* < 0.001, ^##^*P* < 0.01, ^#^*P* < 0.05 as compared to lane 2, ^$$$^*P* < 0.001, ^$$^*P* < 0.01 as compared to lane 6. *IB* Immunoblot; *IP* Immunoprecipitation

PHLPP1 over-expression increased PHLPP1 expression (Fig. 4A), without affecting PHLPP2 expression (Fig. 4B) under MF and MFI condition respectively, further corroborating that PHLPP1 and PHLPP2 do not show compensatory upregulation in expression. Due to PHLPP1 overexpression, there was no change in expression of Akt1, Akt2 or Akt3 under all the conditions tested (Fig. 4C–E). However, phosphorylation of Akt2 (Fig. 4D) and Akt3 (Fig. 4E) was down-regulated by insulin stimulation under MF and MFI conditions, without any effect on Akt1 phosphorylation (Fig. 4C). As observed in PHLPP1 silencing data, there was no change in expression of AS160 under all the conditions tested (Fig. 4F, G). However, activation of AS160 at Ser-588 and Thr-642 was down-regulated by insulin stimulation under MF and MFI conditions, respectively. The effect was more pronounced under over-expressed condition as compared to silencing in the resistant cells, which may be attributed to elevated endogenous expression of PHLPP1 in inulin resistant neuronal cells. PHLPP2 over-expression increased PHLPP2 expression (Fig. 5A), without affecting PHLPP1 expression (Fig. 5B) under MF and MFI condition, respectively. Post PHLPP2 over-expression, there was no change in expression of Akt1 (Fig. 5C), Akt2 (Fig. 5D) or Akt3 (Fig. 5E) under all the conditions tested. However, phosphorylation of Akt1 (Fig. 5C) and Akt3 (Fig. 5E) was down-regulated by insulin stimulation under MF and MFI conditions, without any effect on Akt2 phosphorylation (Fig. 5D). Post PHLPP2 over-expression, there was no change in expression of AS160 under all the conditions tested (Fig. 5F, G). However, activation of AS160 at Ser-588 and Thr-642 was down-regulated by insulin stimulation under MF and MFI conditions, respectively. This PHLPP1 and PHLPP2 over-expression data in insulin sensitive and resistant N2A cells, and how it affects downstream substrates has been summarized in Additional file 6: Table S2.

To determine whether the above-mentioned effects were not cell or species specific, we tested the effect of PHLPP1 and PHLPP2 over-expression on expression and phosphorylation of AS160 (Thr-642 and Ser-588 sites), under MF and MFI condition in differentiated SHSY-5Y cells (Additional file 3: Fig. S3). The effects followed the similar trend as in N2A (data has been consolidated in Additional file 6: Table S3). Thus, data shows that PHLPP regulate Akt isoforms affecting AS160 in neuronal insulin signaling and resistance in an isoform specific manner.

### Effect of PHLPP1 and PHLPP2 silencing and over-expression on glucose uptake in insulin-resistant neuronal cells

**Fig. 6:**
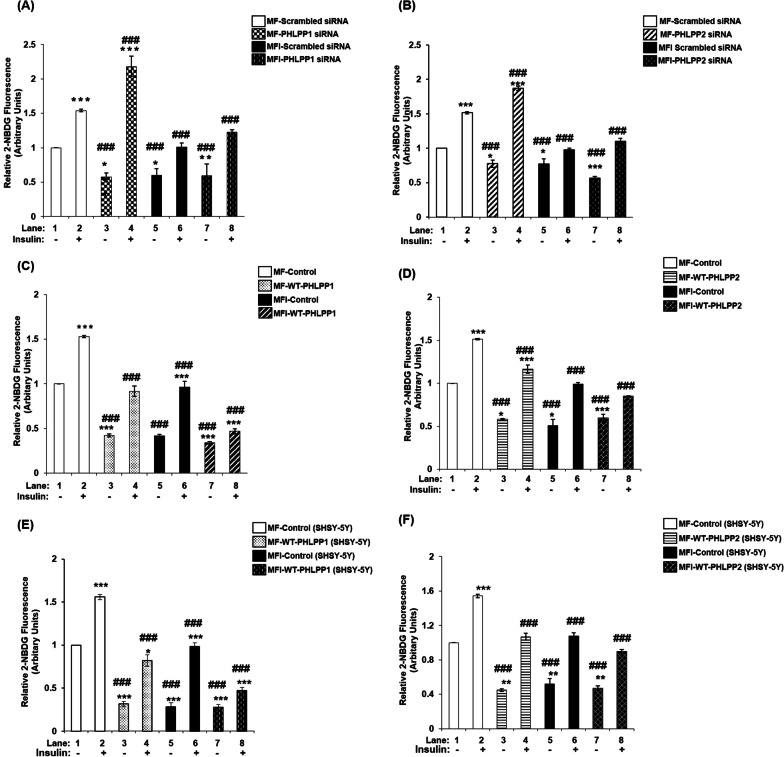
Effect of PHLPP isoform specific silencing or over-expression on glucose uptake. Three days post-proliferation, PHLPP1 or PHLPP2 was silenced or over-expressed using isoform specific siRNA or over-expression plasmid respectively, as indicated. **(A–D)** N2A cells or **(E–F)** SHSY-5Y were differentiated in serum-free medium in the absence of (MF) or chronic presence of 100 nM insulin (MFI) for 3 days. Differentiated cells were serum starved for 2 h, followed by 100 nM insulin for 30 min. Uptake of 2-NBDG was then measured. Bar represents relative change in uptake of 2-NBDG. Experiments were executed three times and a representative result is shown. Data expressed are mean ± SE. ***P < 0.001, **P < 0.01, *P < 0.05 compared to lane 1, ^**###**^*P* < 0.001, ^**##**^*P* < 0.01, ^**#**^*P* < 0.05 as compared to lane 2. *A.U* Arbitrary Units

Having observed PHLPP isoform specific regulation of Akt isoforms and AS160, we sought out to determine the effect of this on neuronal glucose uptake, if any. PHLPP1 or PHLPP2, silenced or over-expressed differentiated N2A cells were stimulated with or without insulin and subjected to glucose uptake assay. PHLPP1 silencing caused an increase of insulin stimulated 2-NBDG uptake by 42% (Fig. 6A, lane 2 vs. lane 4), and 21% (Fig. 6A, lane 6 vs. lane 8) respectively, when compared MF to respective MFI condition. Similarly, PHLPP1 over-expression caused a decrease (Fig. 6C). PHLPP2 silencing caused an increase of 23% (Fig. 6B, lane 2 vs. lane 4), and 12% (Fig. 6B, lane 6 vs. lane 8) respectively, in insulin stimulated 2-NBDG uptake when compared MF to MFI condition. Similarly, PHLPP2 over-expression caused a decrease (Fig. 6D). This PHLPP isoform specific silencing data has been consolidated in Additional file 6: Table S1, and over-expression data in Additional file 6: Table S2. We conducted the same tests in differentiated SHSY-5Y cells and the effects followed a similar trend (Fig. 6E, F). The data has been consolidated in Additional file 6: Table S3. These data reports PHLPP isoform specificity in regulating glucose uptake in neuronal insulin signaling and resistance.

**Fig. 7:**
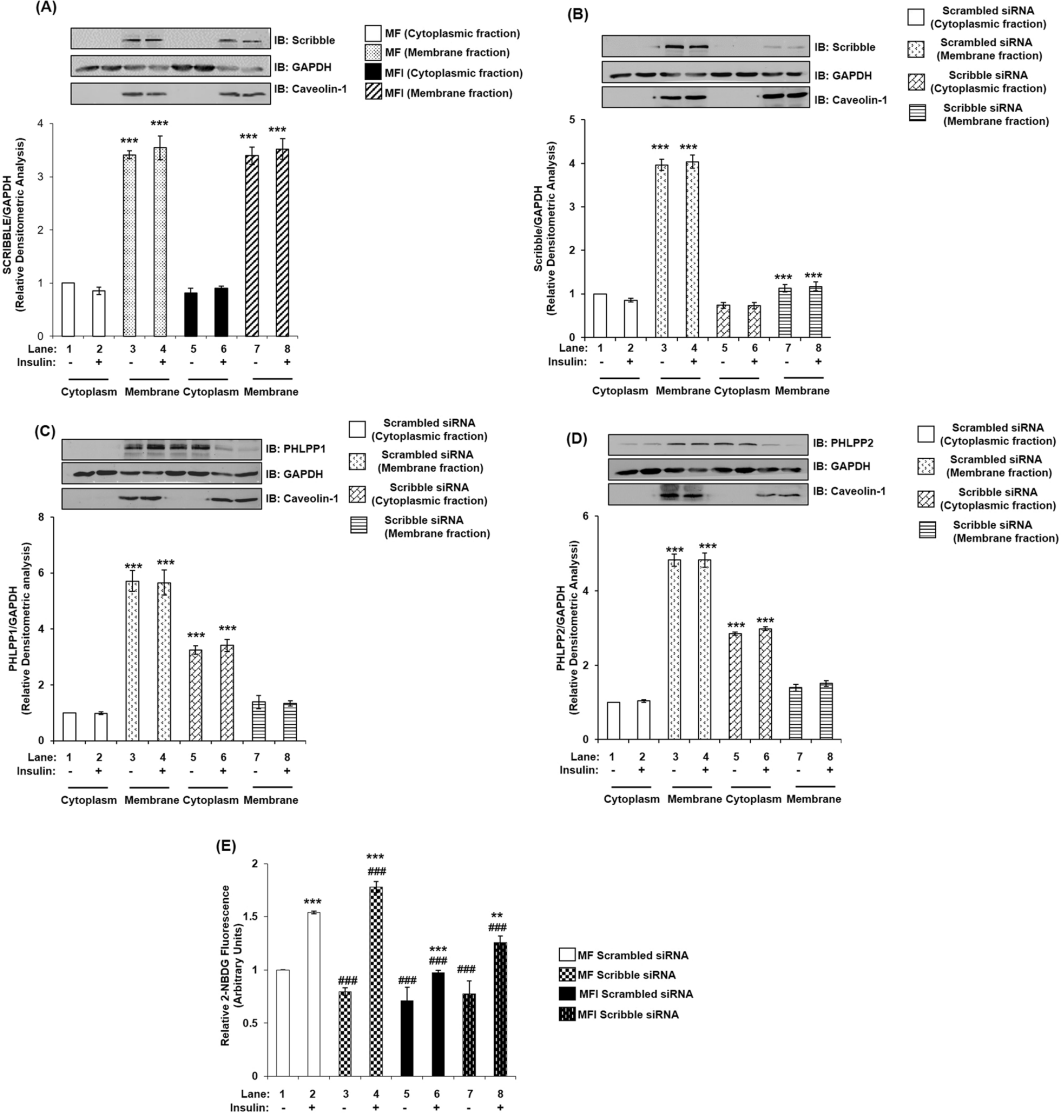
Effect of Scribble silencing on Scribble, membrane localization of PHLPP isoforms, and neuronal glucose uptake under insulin sensitive and insulin resistant condition in neuronal cells (N2A). N2A cells were differentiated in serum-free medium in the absence of (MF) or chronic presence of 100 nM insulin (MFI) for 3 days. Three days post-differentiation, membrane and cytosol fraction were isolated and subjected to western blotting, followed by probing with relevant primary antibodies. **A** Bar represents relative change in Scribble when probed with anti-Scribble antibody. **(B-D)** Three days post-proliferation, Scribble was silenced using Scribble specific siRNA. N2A cells were differentiated in serum-free medium in the absence of insulin (MF) for 3 days. Three days post-differentiation, membrane and cytosol fraction were isolated and subjected to western blotting, followed by probing with relevant primary antibodies. **B** Bar represents relative change in Scribble when probed with anti-Scribble antibody. **C** Bar represents relative change in PHLPP1 probed with anti-PHLPP1 antibody. **D** Bar represents relative change in PHLPP2 probed with anti-PHLPP2 antibody. **E** Three days post-proliferation, N2A cells were transfected with Scribble specific siRNA and then differentiated under MF MFI condition for 3 days. Differentiated N2A cells were serum starved for 2 h, followed by 100 nM insulin for 30 min. Uptake of 2-NBDG was then measured. Experiments were executed three times and a representative result is shown. Data expressed are mean ± SE. ***P < 0.001, **P < 0.01, *P < 0.05 compared to lane 1, ^###^*P* < 0.001, ^##^*P* < 0.01, ^#^*P* < 0.05 as compared to lane 2. *IB* Immunoblot; *A.U* Arbitrary Units

Effect of insulin on expression and membrane localization of Scribble in insulin signaling and insulin resistant neuronal cells. Insulin-sensitive (MF) and insulin-resistant (MFI) N2A cells were stimulated with or without insulin (100 nM, 30 min), lysed and fractionated into cytoplasmic and membrane fractions [1]. Lysates were subjected to western immunoblotting probed with anti-Scribble antibody. Scribble was found to be localized in the membrane fraction only as compared to the cytoplasmic fraction (Fig. 7A), and the expression was not regulated by insulin stimulation (Fig. 7A). Expression of Caveolin-1 is considered as a plasma membrane specific marker and the degree of purity of membrane fractions [31]. Expression of Caveolin-1 was only observed in membrane fraction as compared to cytoplasm (Fig. 7A–D). Scribble was also observed in the membrane fractions and in similar quantity in MF and MFI and their (trans)location to the membrane was irrespective of insulin stimulation. This was determined by using GAPDH as a loading control, to perform densitometric analyses, because GAPDH is present in both “cytoplasm” and “membrane” [32], as only membrane-specific markers cannot be used for densitometry analysis of “cytoplasm” fractions. Data shows that Scribble is present in N2A cells and localized at the membrane.

### Effect of Scribble silencing on membrane translocation of PHLPP1 and PHLPP2

Scribble was silenced and its effect on membrane localization was tested on PHLPP1 and PHLPP2, with or without insulin stimulation. 100 nM siRNA was required to silence 70% of Scribble (Additional file 4: Fig. S4). Cells were lysed and fractionated into cytoplasmic and membrane fractions, and lysates were subjected to western immunoblotting probed with anti-Scribble, anti-PHLPP1, anti-PHLPP2 antibody, as and when indicated.

Consistent with the previous result (Fig. 7A), insulin stimulation did not affect Scribble expression, and Scribble was found to be localized on the membrane only as compared to the cytoplasm. Scribble silencing decreased its expression by 70% (Fig. 7B, lane 3 vs. lane 7, lane 4 vs. lane 8) in the membrane fraction. Under control (scrambled siRNA transfected) condition, PHLPP1 was localized on the membrane only (Fig. 7C, lane 3, 4). As a result of Scribble silencing, PHLPP1 translocated from the membrane to the cytoplasm by 60% (Fig. 7C, lane 5 vs. lane 7, lane 6 vs. lane 8). Similarly, under control (scrambled siRNA transfected) condition, PHLPP2 was also localized on the membrane only, with limited expression in the cytoplasm (Fig. 7D lane 3, 4). Scribble silencing caused PHLPP2 translocation from the membrane to the cytoplasm by 50% (Fig. 7D, lane 5 vs. lane 7, lane 6 vs. lane 8). Data shows Scribble determines cellular localization of both PHLPP1 and PHLPP2. This is in coherence with previous study by Li et al., where they reported that in colorectal cancer cells Scribble silencing affected membrane localization of PHLPP isoforms, affecting PHLPP1 more than PHLPP2 [18].

### Effect of Scribble silencing on neuronal glucose uptake in insulin signaling and insulin resistant neuronal cells

Scribble has previously been reported to play varied roles as a tumor suppressor [29, 30] but its possible role in glucose uptake in insulin signaling has not been reported. We proceeded to test its effect on neuronal glucose uptake in insulin signaling and insulin resistance. Scribble silenced differentiated N2A cells were stimulated with or without insulin and subjected to glucose uptake assay. Its silencing caused a increase of 16% (Fig. 7E, lane 2 vs. lane 4) and 29% (Fig. 7E, lane 6 vs. lane 8) respectively, in insulin stimulated 2-NBDG uptake when compared MF to MFI condition, respectively. Data establishes Scribble as an important modulator of neuronal glucose uptake under insulin sensitive and resistant condition.

## Discussion

### Differential expression of PHLPP isoforms in neuronal insulin resistance

In the present study, we report differential increase in expression of both PHLPP1 and PHLPP2, in hyperinsulinemia mediated insulin resistance in two neuronal cell lines in vitro*,* with or without insulin stimulation (Fig. 1A–D). We observed that resistance caused due to high-fat-diet in mice whole brain tissue lysate also caused a similar differential increase in PHLPP1 and PHLPP2 expression (Fig. 1E, F). Interestingly, in both the cases, resistance mediated expression of PHLPP1 was significantly more as compared to PHLPP2. This study reports differential increase in expression of both PHLPP1 and PHLPP2 in insulin sensitive and insulin resistant condition, in a neuronal system.

In insulin-dependent tissue systems (e.g., skeletal muscles, adipocytes, hepatocytes and pancreases), there are paradoxical data regarding PHLPP isoform specific expression under pathological conditions like diabetes and obesity. Cozzone et al. reported impaired insulin signaling in skeletal muscles of T2D patients due to deregulated serine phosphorylation of Akt2 and Akt3 (and threonine phosphorylation of Akt1) [9]. This Akt2 and -3 serine specific deregulation was attributed to elevated PHLPP1 mRNA (but not PHLPP2 mRNA). Andreozzi et al. demonstrated that in skeletal muscles and abdominal subcutaneous adipose tissue samples of morbidly obese participants, PHLPP1 expression was elevated and inversely related to Akt serine phosphorylation [10]. Interestingly, these studies negate any involvement of PHLPP2 under pathological disorders [9, 10]. Wu et al. and Kim et al. reported elevated PHLPP2 from adipose tissue of obese leptin receptor-deficient db/db mice and HFD fed mice respectively [11, 33]. The study by Kim et al. comparing C57BL/6 J mice, obese db/db mice to liver-specific PHLPP2 knockout mice under normal chow or high fat diet reported PHLPP2 knockout induced lipogenesis [34]. Hribal et al. reported elevated PHLPP1 and PHLPP2 expression in pancreatic β cells post chronic exposure to high glucose concentrations [35]. Drawing a parallel between their study and ours, both studies report differential increase in PHLPP1 and PHLPP2 levels. While our study reports more elevated expression of PHLPP1 than PHLPP2 post chronic insulin stimulation in neuronal cells, their study reports PHLPP2 expression to be more elevated than PHLPP1 post chronic glucose exposure in pancreatic β-cells. Lupse et al. also reported upregulated PHLPP1 and PHLPP2 in human islets and β-cells under glucotoxic conditions in vitro and in islets from diabetic mouse models and in patients with T2D [13]. Thus, our study reports differential increase in expression of both PHLPP1 and PHLPP2 (PHLPP1 > PHLPP2) in insulin resistant neuronal cells and in high-fat-diet mice brain lysate.

### PHLPP isoforms regulate Akt isoforms differentially in neuronal insulin signaling and insulin resistance

Brognard et al. were the first to report that PHLPP1 specifically regulates Akt2 and Akt3, while PHLPP2 regulates Akt1 and Akt3 in H157 and Hs578Bst cell lines [2]. Pie et al. further consolidated PHLPP isoform specific regulation of Akt isoforms in 293 T cells. Nitsche et al. reported similar regulation in human PDAC and normal pancreatic tissue samples in vivo as well as in PaCa-2 and PANC-1 cell line in vitro*,* that PHLPP1 regulates phosphorylation of Akt2 and PHLPP2 regulates Akt1 specifically (Akt3 was not included in this study). Paradoxically, Miyamoto et al. reported PHLPP1 (but not PHLPP2) silencing in neonatal rat ventricular myocytes (NRVMs) and in adult mouse ventricular myocytes affected Akt serine phosphorylation. They reported comparable increase in phosphorylation and catalytic activity of both Akt1 and Akt2 (Akt3 was not addressed in this study). They attributed these deviations to terminally differentiated cardiomyocytes of their study, in contrast to previous studies done in breast and colorectal cancer cells [2, 3]. Moc et al. in mice heart reported PHLPP1 knockout increased Akt1 activity following transverse aortic constriction pressure (but not Akt2), increasing cardiomyocyte size and leading to pathological hypertrophy. These studies depict PHLPP isoform specificity can be tissue dependent. In neuronal system, there is only one previous report in mice brain where all three isoforms of Akt have been studied downstream of both PHLPP isoforms. Chen et al. reported PHLPP1 knockout increased Akt activity, without any effect on Akt isoform expression. However, Akt isoform specific phosphorylation post PHLPP1 knockout was not addressed in this study. Therefore, our study is a holistic study to report how both isoforms of PHLPP regulate three isoforms of Akt under insulin sensitive and resistant neuronal condition. Previously, Cozzone et al. had correlated decreased Akt2 and -3 serine phosphorylation in skeletal muscles of T2D patients, and attributed it to increased PHLPP1 mRNA levels (with no effect on PHLPP2 mRNA and corresponding no effect on Akt1 serine phosphorylation). Our present study comprehensively associates differential increase in protein expression of PHLPP1 and PHLPP2 with concomitant decrease in serine phosphorylation of Akt in isoform specific manner.

### PHLPP isoforms control Akt isoform mediated regulation of AS160 and glucose uptake in neuronal insulin signaling and insulin resistance

AS160 is a RabGAP (Rab GTPase-activating protein), regulating GLUT4 translocation from the GLUT4 Storage Vesicles (GSVs) to the plasma membrane. Post insulin stimulation, activated Akt phosphorylates AS160 at Ser-588 and Thr-642 sites, hence inactivating it, allowing GLUT4 translocation and glucose uptake to occur [36–39]. In insulin dependent tissue systems (skeletal muscles, adipocytes), only Akt2 has been reported to regulate AS160 and contribute to glucose uptake [36]. However, we had previously established that all Akt isoforms differentially regulate phosphorylation of AS160 at both serine and threonine sites, with Akt2 contributing the most, followed by Akt3 and Akt1 in insulin sensitive and resistant condition [1]. Having established PHLPP isoform specificity in neuronal insulin sensitive and resistant cells, and their specific regulation of Akt isoforms in the present study, we extended the study to understand the regulation of AS160 as well. These data (Fig. 2, 3, 4, 5F, G) have been consolidated in Additional file 5, 6: Table S1, S2, S3 and Fig. S5. An important observation here is, while both PHLPP isoforms regulate phosphorylation of AS160, PHLPP1 affected AS160 phosphorylation more than PHLPP2. This may be attributed to differential regulation of AS160 by Akt isoforms. Although all Akt isoforms regulate AS160 phosphorylation, Akt2 affects it the most, followed by Akt3 and then Akt1 [1]. Since PHLPP1 regulates Akt2 and Akt3, it affects AS160 more than PHLPP2, which regulates Akt1 and Akt3. Previously, PHLPP isoforms have been reported to regulate amplitude and specificity of Akt substrates, aiding in Akt isoform specificity [2]. However, even though AS160 has been studied as a downstream target of Akt, till date there has been no study of possible regulation of AS160 expression and phosphorylation by PHLPP isoforms. Our study reports PHLPP isoform specific regulation affecting AS160 phosphorylation in any system.

**Fig. 8:**
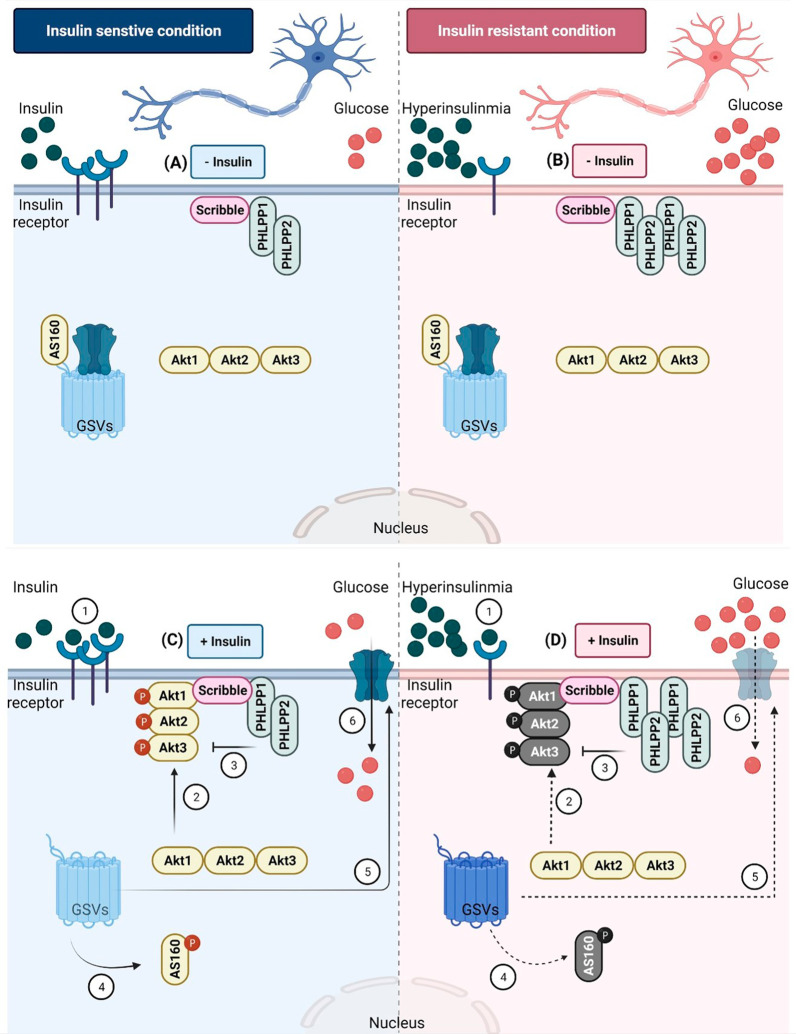
Schematic diagram depicting role of PHLPP isoforms in regulating neuronal insulin signaling. **A** In an insulin sensitive, under unstimulated condition, PHLPP1 is localized on the membrane, while PHLPP2 is on the membrane as well as cytoplasm. Akt isoforms are present in the cytoplasm. AS160 binds to GSVs (GLUT4 Storage Vesicles) and tethers GLUT4. This does not allow GLUT4 exocytosis under basal conditions. **B** In an insulin resistant, unstimulated condition, hyperinsulinemia occurs due to defects in insulin signaling. PHLPP isoforms expression is elevated on the membrane. Scribble, the scaffolding protein holds PHLPP isoforms in place. **C** In an insulin sensitive, insulin stimulated condition, Akt translocates to plasma membrane in an isoform specific insulin dependent way (1), getting phosphorylated there (2). In neuronal system, all Akt isoforms translocate in the order Akt2 > Akt3 > Akt1. PHLPP1/2 are present on the membrane, constant flux of phosphorylation and dephosphorylation (3). An activated Akt phosphorylates AS160, hence inactivating it. Phosphorylated and thus inactivated AS160 translocated to cytoplasm (4), promoting GLUT4 exocytosis (5), and allowing glucose uptake (6). **D** In an insulin resistant, insulin stimulated condition, hyperinsulinemia triggers insulin receptor down-regulation, not allowing Akt isoform specific translocation to the plasma membrane (1). Elevated PHLPP isoforms dephosphorylate Akt isoforms remaining on the membrane (2). This leads to inadequate phosphorylation (3), leading to Akt’s inability to phosphorylate AS160 (4). Unphosphorylated, thus, active AS160 continues tethering GLUT4 to GSVs, not allowing GLUT4 exocytosis (5), hence affecting neuronal glucose uptake (6). (Created with BioRender.com)

With regard to glucose uptake, we observed differential isoform-specific regulation wherein PHLPP1 and PHLPP2 regulate glucose uptake post insulin stimulation, under insulin sensitive and resistant conditions. An interesting point to note is, under both insulin sensitive and resistant condition, PHLPP1 affected neuronal glucose uptake more as compared to PHLPP2, primarily attributed to PHLPP isoform specificity from Akt isoforms to AS160 regulation, ultimately affecting glucose uptake. Previously, Andreoozi et al. had reported that overexpression of PHLPP1 abolished the effect of insulin on glucose uptake in L6 cells. Xiong et al. had reported both PHLPP isoforms silencing elevated glucose metabolism via Akt in SW480, DLD1 and Caco2 cells. However, this study did not address role of Akt isoforms or AS160 in glucose uptake. We report PHLPP isoform specific regulation of glucose uptake via AS160 affecting in insulin sensitive and resistant condition, in neuronal cells. This pathway has been explained in Fig. 8 and Additional file 5: Fig. S5.

### Scribble, the scaffolding protein, mediate PHLPP isoform specific regulation

Our data suggest Scribble regulates cellular localization of both isoforms of PHLPP, allowing PHLPP to exercise its downstream isoform specific effects (Fig. 7C, D). In contrast to a study by Li et al., which reported Scribble binding and affecting redistribution of only PHLPP1, we report Scribble silencing affecting both isoforms of PHLPP (PHLPP2 to a lower extent than PHLPP1). Li et al. have previously demonstrated that dissociation of PHLPP1 from the membrane, due to Scribble silencing, impaired its ability to dephosphorylate Akt. Interestingly, in our study, under Scribble silenced condition neuronal glucose uptake displayed a modest but significant increase post insulin stimulation under insulin sensitive and resistant condition (Fig. 7E). Thus, Scribble may be proposed to have an insulin sensitizing effect, as Scribble silencing affect PHLPP localization, which decreases its interaction with Akt isoforms, increasing AS160 and GLUT4 translocation, ultimately increasing glucose uptake. This increase in glucose uptake was observed under insulin sensitive and resistant condition (Fig. 7E), further ascertaining a possible role of Scribble as an insulin sensitizer protein. However, a strong possibility of other proteins constituting scaffold play a role in facilitating proximity to PHLPP and Akt, resulting into much higher contribution to glucose uptake, as compared to Scribble, cannot be ignored. The importance of the functional interaction between PHLPP and Scribble has previously been established in tumor progression in colorectal cancer cells [18]. Scribble silencing affects the subcellular localization of PTEN as well, activating the AKT-mTOR-S6 kinase signaling pathway and promoting mammary tumorigenesis in breast cancer [21]. Troyanovsky et al. proposed that Scribble and PP1 phosphatase make a complex near the basolateral cell cortex, where PP1 is maintained in an inactive form. In absence of Scribble, PP1 is constantly active, leading to chaotic dephosphorylation of various downstream targets. Even though Scribble has been implicated in regulating cellular localization of various phosphatases (PHLPP, PTEN, PP1) in cancer cells, they have even been studied extensively in insulin signaling as well, no study has yet reported Scribble’s function with reference to glucose uptake and its possible metabolic and pathological implications in terms of insulin resistance. Our study elucidates how Scribble affects cellular localization of PHLPP isoforms, hampering its ability to dephosphorylate Akt isoforms, ultimately affecting neuronal glucose uptake and insulin resistance.

## Conclusions

Our study reports isoform specific role of both PHLPP isoforms in regulating neuronal insulin signaling and resistance. We find the following: (a) elevated expression of both PHLPP1 and PHLPP2 in insulin resistant neuronal cells and high-fat-diet fed mice whole brain lysate.; (b) PHLPP1 regulates serine phosphorylation of Akt2 and Akt3, and PHLPP2 regulates serine phosphorylation of Akt1 and Akt3 in neuronal cell insulin signaling; (c) PHLPP1 regulates serine phosphorylation of Akt2 and Akt3, and PHLPP2 regulates serine phosphorylation of Akt1 and Akt3 in insulin resistant neuronal cells; d) both PHLPP isoforms regulate three Akt isoforms, extending the regulation to AS160 (e) both the isoforms of PHLPP regulate glucose uptake in insulin sensitive and insulin resistant neuronal cells (f) PHLPP isoform specificity is mediated by Scribble, which mediates cellular localization of isoforms, regulating neuronal insulin signaling; (g) a novel role of Scribble in regulating glucose uptake in neuronal cells. All data points to a differential and independent role of PHLPP isoforms in neuronal insulin signaling and insulin resistance. These observations are significant and insightful in understanding insulin signaling and insulin resistance. Further, this study in neuronal insulin resistance, which is one of the hallmarks of several neurodegenerative diseases, may help in taking a step forward in solving problems associated with a Type 3 diabetes, diabetes complications and neurodegenerative disorders.

## Materials and methods

### Materials

Minimum essential media (MEM), Dulbecco’s Minimum essential media (DMEM), foetal bovine serum (FBS), trypsin-EDTA, Opti-MEM, 2-(*N*-(7-nitrobenz-2-oxa1,3-diazol-4-yl) amino)-2 deoxy glucose (2-NBDG) (Cat. No. N13195), Lipofectamine 2000 (Cat. No. 11668019), anti-PHLPP1 antibody (Cat. No. 07-1341), anti-Scribble antibody (Cat. No. PA5-23628), anti-Caveolin-1 antibody (Cat. No. PA1-064) and anti-Akt3 antibody (Cat. No. 41700) were procured from Thermo Fisher Scientific Inc. (USA). MCDB 201 medium, nutrient mixture F-12 Ham, albumin from bovine serum (cell culture grade), anti-GAPDH antibody (Cat. No. 9545), anti-PHLPP2 (Cat. No. A300-661A-T) and dimethyl sulfoxide (DMSO) were purchased from Sigma-Aldrich Co. (USA). Anti-phospho-Akt (serine-473) (Cat. No. 4058), anti-phospho-Akt1(Serine-473) (Cat. No. 9018), anti-phospho-Akt2 (Serine-474) (Cat. No. 8599), anti-Akt1 antibody (Cat. No. 2938), anti-Akt2 antibody (Cat. No. 3063), anti-phospho-AS160 (serine-588) (Cat. No. 8730), anti-phospho-AS160 (threonine-642) (Cat. No. 8881), anti-AS160 antibody (Cat. No. 2670), IgG conformational (Cat. No. 3678) were purchased from Cell Signalling Technology Inc. (USA). Protein A/G agarose beads (Cat no. sc: 2003) were purchased from Santa Cruz Biotechnology (USA). Recombinant insulin (Cat. No. 407709) was purchased from Calbiochem (Germany).

### Cell culture

N2A (mice neuroblastoma cell line) and SHSY-5Y (human neuroblastoma cell line) cells were proliferated in MEM, and equal mixture of DMEM and Ham’s F12 medium respectively, supplemented with 10% FBS, and antibiotics-penicillin 100 IU/ml and streptomycin 100 mg/ml, at 37 °C in 5% CO_2_. Insulin resistance in N2A and SHSY-5Y cells was generated as reported earlier from our laboratory [1, 26, 27, 29, 30]. Briefly, cells were kept in an equal mixture of two serum-free media (MCDB 201 medium and nutrient mixture F-12 Ham) in the absence (MF; insulin sensitive) or in chronic presence of 100 nM insulin for 3 days (MFI; insulin resistant) with 2% DMSO (N2A cells) or 10 µM Retinoic acid (SHSY-5Y cells). Media were replaced after every 12 h. Differentiated neuronal cells were subjected to 30 min insulin stimulation as per standard assay conditions as reported previously from other as well as from our laboratory.

### Gene silencing by siRNA transfection

PHLPP isoforms in the cells were silenced as previously reported [1]. To select the specific siRNA among 4 different siRNAs against each isoform provided by the manufacture (Qiagen), specific siRNA for each isoform was chosen based on the one that provided maximum silencing (Additional files 1, 2, 4: Figs. S1A, S2A, S4A). Selected isoform specific siRNA sequences were: PHLPP1:5′-CTGGAGGAGTTTGATACAGCA-3’; PHLPP2:5’-CAGCGGCTTATACAGATTGTA-3’; Scribble:5’-CTGGAGGGACTTACCCACCTA-3’. Selection of specific siRNA was followed by optimization of dose of selected siRNA (Additional files 1, 2, 4: Figs. S1B, S2B, S4B). Scrambled (non-specific) and PHLPP isoform specific or Scribble specific siRNAs were transfected in Opti-MEM using Lipofectamine 2000. Six hours post transfection, Opti-MEM was changed to respective proliferation media depending on the cell line used. Twelve hours post media change, cells were subjected to differentiation as explained above.

### Plasmid DNA transfection using PHLPP isoform specific plasmids

PHLPP1 specific construct were a kind gift from Dr. Kishore Parsa, Dr. Reddy’s Institute of Life Sciences (DRILS), Hyderabad, India. PHLPP2 specific construct were purchased from Addgene [(#22403)]. Specific plasmid constructs were transfected in the cells as previously reported. Briefly, N2A or SHSY-5Y cells were transiently transfected with either specific construct or control (respective empty backbone) constructs in Opti-MEM using Lipofectamine 2000 according to manufacturer's instructions.

### Immunoprecipitation

Because a specific phospho-Akt3 antibody was not available to measure phosphorylation levels of Akt3, Akt3 protein was immunoprecipitated using Akt3 specific antibody (Thermo, PA-41700), and probed with Anti-phospho-Akt (serine-473) (CST, Cat. No. 4058)**.** Anti-IgG conformational antibody (CST, Cat No. 3678) was used to mask remaining IgG bands. Immunoprecipitation was performed as previously reported [1].

### Cell lysis and immunoblotting

After differentiation, cells were stimulated with or without 100 nM insulin for 30 min as indicated above. Cells were lysed in lysis buffer as described previously from our laboratory for 30 min at 4 °C [1, 26–30]. Supernatants were collected and protein was estimated by Bicinchoninic Acid (BCA) method [40]. Equal amount of protein of all the samples was then resolved on SDS-PAGE, followed by Western immunoblotting. For quantification of immunoblots, Quantity One 1-D analysis software (Bio-Rad Laboratories, Inc.) was used as described previously [1].

### Membrane fractionation

N2A cells were differentiated in 100 mm tissue culture plates. After respective treatment, cells were lysed in lysis buffer (20 mM Tris, pH 7.5; 250 mM sucrose; 2 mM EDTA; 2 mM EGTA; 1 mM PMSF; 10 µg/ml aprotinin and leupeptin), as reported earlier from our laboratory for 5 min [41]. Lysate was homogenized for 10 min with 30 strokes in Glass homogenizer at 4 °C. Lysates were subjected to ultra-centrifugation, at 200,000×*g* for 1 h. Supernatant was collected and termed as ‘Cytosolic fraction’ and pellet was re-suspended in lysis buffer with 1% NP40 (20 mM Tris, pH 7.5; 1% NP-40; 150 mM NaCl; 1 mM EDTA; 1 mM EGTA; 1 mM PMSF; 10 µg/ml aprotinin and leupeptin) at 4 °C for 30 min. Centrifuged supernatant was collected and termed as ‘Membrane fraction’. Presence of membrane-specific marker like Caveolin-1 was tested and GAPDH was used as a loading control in all membrane translocation experiments [32].

### Mice whole brain tissue lysis and immunoblotting

Mice whole brain were lysed in a homogenizer in lysis buffer at 4 °C for 15 min. Mice whole brain tissues were homogenized as described previously [1]. Supernatants were collected and protein was estimated by Bicinchoninic acid test. All experiments were performed following the guidelines prescribed by CPCSEA with the approval of the Internal Animal Ethics Committee, Visva-Bharati (IAEC/VB/2017/01).

### Glucose uptake assay

Glucose uptake assays were performed as described previously from our laboratory [1, 27, 42]. Briefly, differentiated N2A and SHSY-5Y were washed and subjected to glucose starvation for 2 h. After respective treatment, cells were treated with 50 μM NBDG for 1 h and lysed in buffer containing 20 mM Tris–HCl pH 7.4, 40 mM KCl, 1% sodium deoxycholate and 1% NP-40 for 15 min at 25 °C with gentle shaking. Cells were then scraped off and centrifuged at 13,000 × g for 20 min at 4 °C. The fluorescence was then measured using a fluorescence spectrophotometer LS55 (Perkin Elmer, USA) at the excitation and emission wavelengths of 485 nm and 535 nm, respectively.

### Replicates and statistical analysis

Data presented depicts three or more biological replicates as mean ± SE. Significance was assessed using an unpaired, two tailed t test and p values indicated in the Figures. Differences were considered significant at *p* < 0.05.

## Supplementary Information

**Additional file 1**. Effect of PHLPP1 silencing on N2A cells.

**Additional file 2**. Effect of PHLPP2 silencing on N2A cells.

**Additional file 3**. Effect of PHLPP 1 or PHLPP2 over-expression on AS160 in insulin signaling and insulin resistant condition in neuronal cells (SHSY-5Y).

**Additional file 4**. Effect of Scribble silencing on N2A cells.

**Additional file 5**. Flowchart of PHLPP isoform specific regulation of Akt isoforms, AS160 and glucose uptake in insulin signaling and -resistance in neuronal cells.

**Additional file 6**. Effect of PHLPP1 or PHLPP2 silencing or over-expression on Akt isoforms, AS160 and neuronal glucose uptake under insulin resistant condition in neuronal cells (N2A/SHSY-5Y).

## Data Availability

All the data related to this manuscript is available with the first author and corresponding author.
