# Supplementary material for: PHLPP isoforms differentially regulate Akt isoforms and AS160 affecting neuronal insulin signaling and insulin resistance via Scribble
Source: Cell Commun Signal. 2022 Nov 14;20:179. doi: 10.1186/s12964-022-00987-0 (PMC9664818; doi:10.1186/s12964-022-00987-0)
Supplement: Supplementary file 2 — Additional file 1. Effect of PHLPP1 silencing on N2A cells. [file 12964_2022_987_MOESM2_ESM.docx]

**PHLPP isoforms differentially regulate Akt isoforms and AS160 affecting neuronal insulin signaling and insulin resistance via Scribble.**

Medha Sharma^1^ and Chinmoy Sankar Dey^1^*

**SUPPLEMENTARY FIGURES:**

**ADDITONAL FILE 1: Effect of PHLPP1 silencing on N2A cells.**


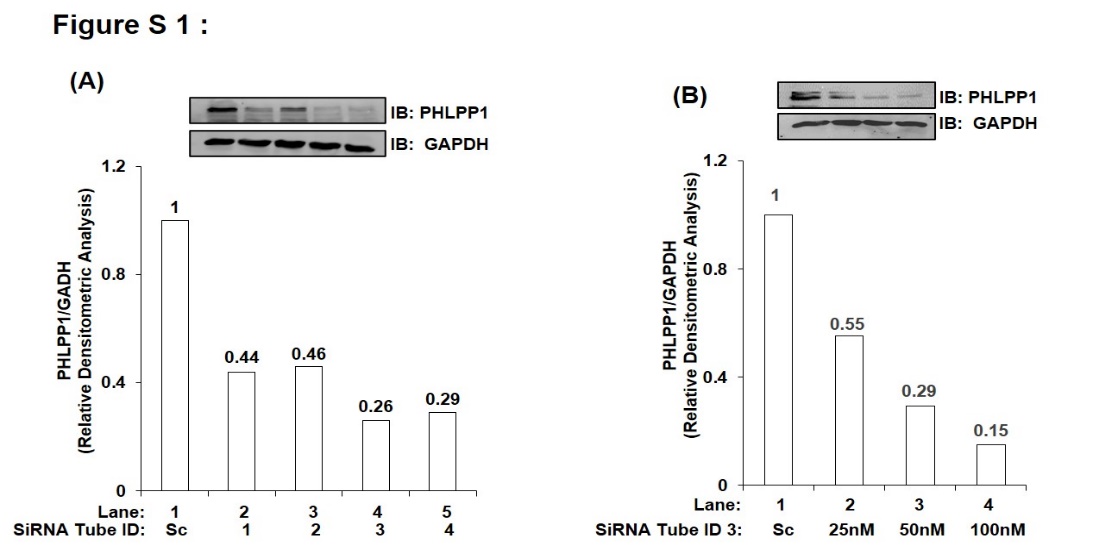


**Fig.S1: Effect of PHLPP1 silencing on N2A cells.** (A) Selection of siRNA. (B) Determination of dose course of selected siRNA at concentration of 25 nM, 50 nM and 100 nM. GAPDH has been used as a loading control. Experiments were executed two times and an average shown.
