# Supplementary material for: PHLPP isoforms differentially regulate Akt isoforms and AS160 affecting neuronal insulin signaling and insulin resistance via Scribble
Source: Cell Commun Signal. 2022 Nov 14;20:179. doi: 10.1186/s12964-022-00987-0 (PMC9664818; doi:10.1186/s12964-022-00987-0)
Supplement: Supplementary file 4 — Additional file 3. Effect of PHLPP 1 or PHLPP2 over-expression on AS160 in insulin signaling and insulin resistant condition in neuronal cells (SHSY-5Y). [file 12964_2022_987_MOESM4_ESM.docx]

**PHLPP isoforms differentially regulate Akt isoforms and AS160 affecting neuronal insulin signaling and insulin resistance via Scribble.**

Medha Sharma^1^ and Chinmoy Sankar Dey^1^*

**SUPPLEMENTARY FIGURES:**

**ADDITIONAL FILE 3: Effect of PHLPP 1 or PHLPP2 over-expression on AS160 in insulin signaling and insulin resistant condition in neuronal cells (SHSY-5Y).**


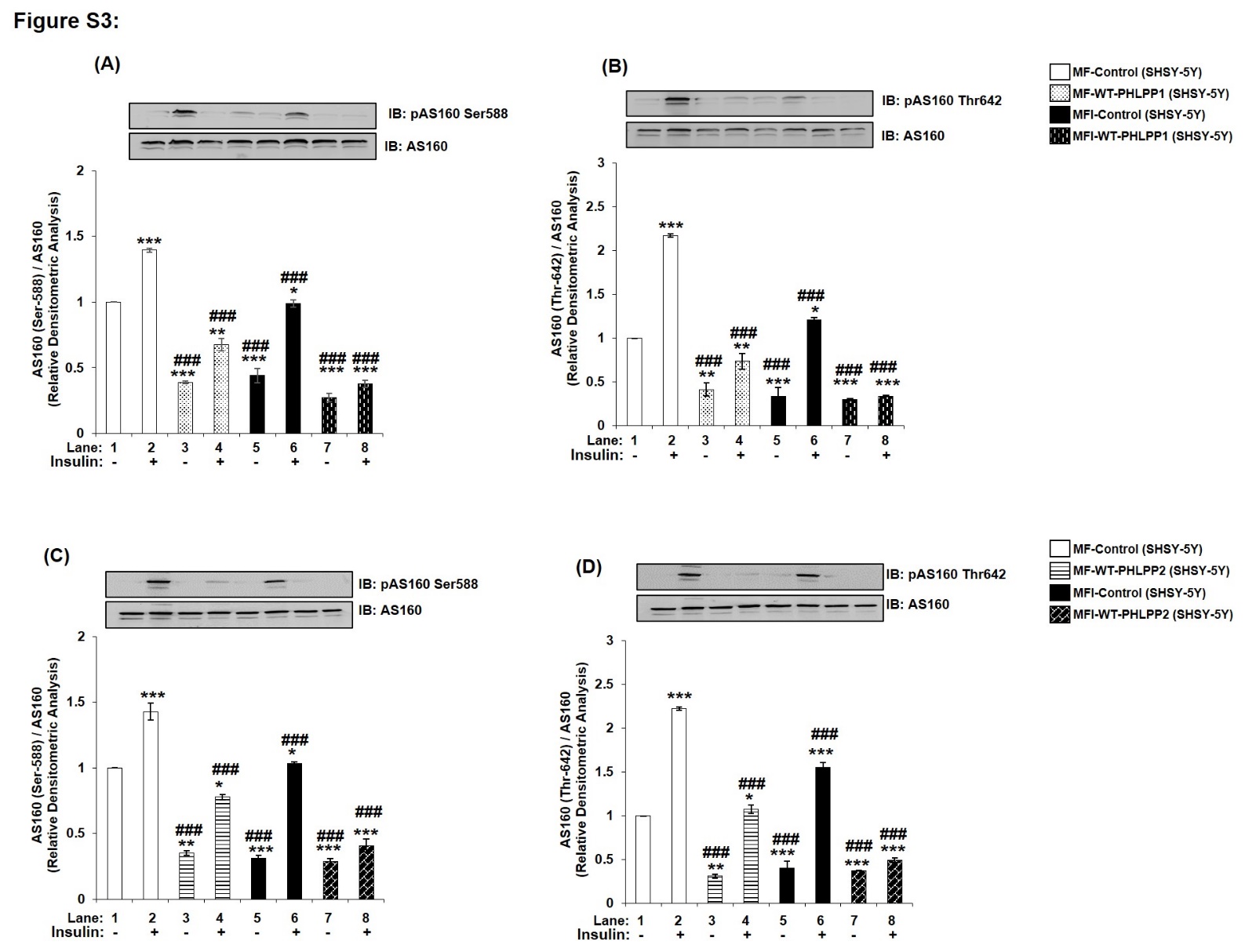


**Fig.S3: Effect of PHLPP 1 or PHLPP2 over-expression on AS160 in insulin signaling and insulin resistant condition in neuronal cells (SHSY-5Y).** Three days post-proliferation, PHLPP1 or PHLPP2 was over-expressed using PHLPP1 or PHLPP2 specific plasmids as indicated. SHSY-5Y cells were differentiated in serum-free medium in the absence of (MF) or chronic presence of 100 nM insulin (MFI) for 3 days. Cells were lysed and subjected to western blotting, followed by probing with relevant primary antibodies. **A, C** Bar represents relative change in pAS160 (Ser-588) when probed with anti-AS160 antibody. **B, D** Bar represents relative change in pAS160 (Thr-642) when probed with anti-AS160 antibody. GAPDH has been used as a loading control. Experiments were executed three times and a representative result is shown. Data expressed are mean ± SE. ****P* < 0.001, ***P* < 0.01, **P* < 0.05 compared to lane 1, **^###^***P* < 0.001, **^##^***P* < 0.01, **^#^***P* < 0.05 as compared to lane 2. *IB* Immunoblot.
