# Supplementary material for: PHLPP isoforms differentially regulate Akt isoforms and AS160 affecting neuronal insulin signaling and insulin resistance via Scribble
Source: Cell Commun Signal. 2022 Nov 14;20:179. doi: 10.1186/s12964-022-00987-0 (PMC9664818; doi:10.1186/s12964-022-00987-0)
Supplement: Supplementary file 6 — Additional file 5. Flowchart of PHLPP isoform specific regulation of Akt isoforms, AS160 and glucose uptake in insulin signaling and -resistance in neuronal cells. [file 12964_2022_987_MOESM6_ESM.docx]

**PHLPP isoforms differentially regulate Akt isoforms and AS160 affecting neuronal insulin signaling and insulin resistance via Scribble.**

Medha Sharma^1^ and Chinmoy Sankar Dey^1^*

**SUPPLEMENTARY FIGURES:**

**ADDITIONAL FILE 5: Flowchart of PHLPP isoform specific regulation of Akt isoforms, AS160 and glucose uptake in insulin signaling and -resistance in neuronal cells.**

**Figure S5:**

**
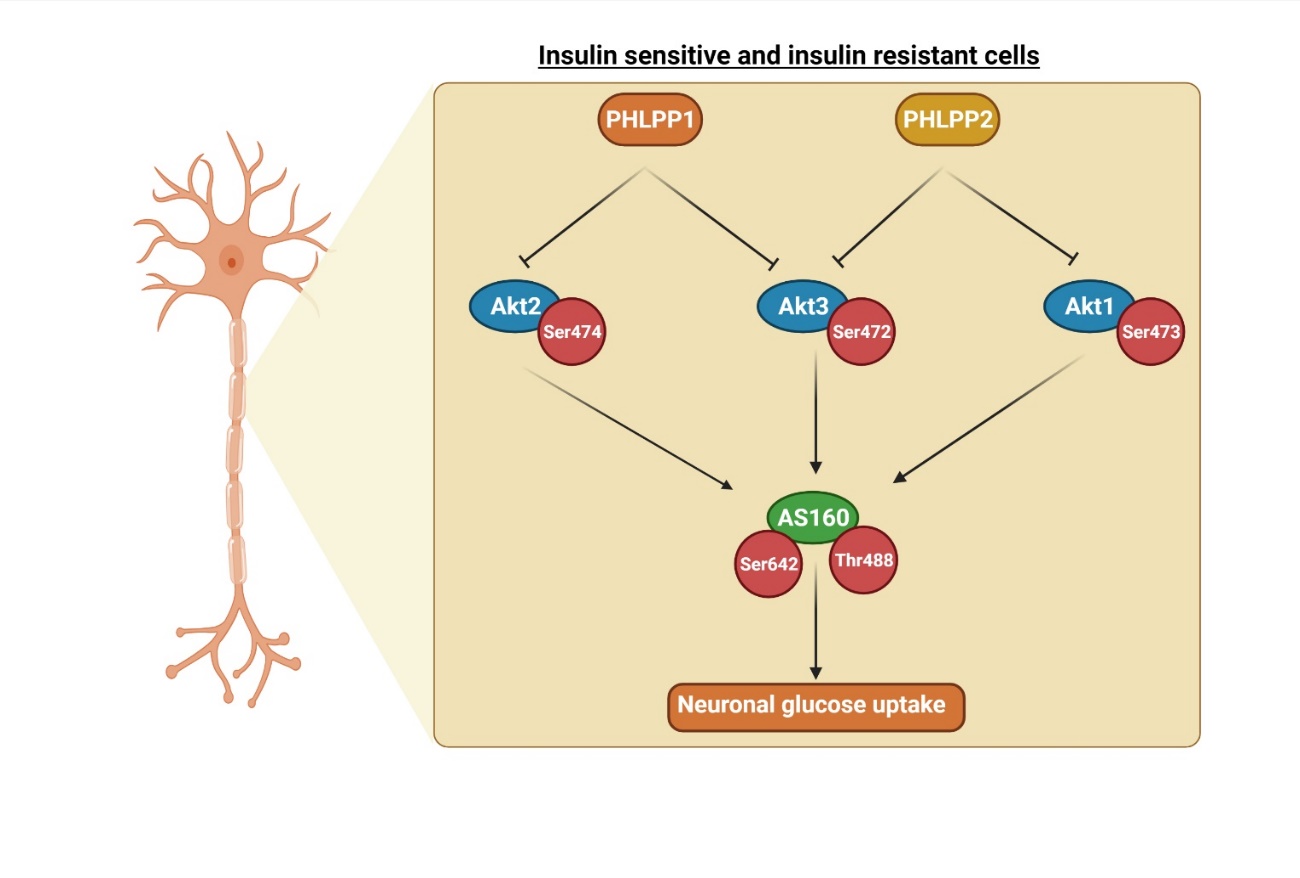
**

**Fig.S5: Flowchart of PHLPP isoform specific regulation of Akt isoforms, AS160 and glucose uptake in insulin signaling and -resistance in neuronal cells.** (Created with BioRender.com)
