# Supplementary material for: PHLPP isoforms differentially regulate Akt isoforms and AS160 affecting neuronal insulin signaling and insulin resistance via Scribble
Source: Cell Commun Signal. 2022 Nov 14;20:179. doi: 10.1186/s12964-022-00987-0 (PMC9664818; doi:10.1186/s12964-022-00987-0)
Supplement: Supplementary file 7 — Additional file 6. Effect of PHLPP1 or PHLPP2 silencing or over-expression on Akt isoforms, AS160 and neuronal glucose uptake under insulin resistant condition in neuronal cells (N2A/SHSY-5Y). [file 12964_2022_987_MOESM7_ESM.docx]

**PHLPP isoforms differentially regulate Akt isoforms and AS160 affecting neuronal insulin signaling and insulin resistance via Scribble.**

Medha Sharma^1^ and Chinmoy Sankar Dey^1^*

**SUPPLEMENTARY TABLES:**

**ADDITONAL FILE 6: Effect of PHLPP1 or PHLPP2 silencing or over-expression on Akt isoforms, AS160 and neuronal glucose uptake under insulin resistant condition in neuronal cells (N2A/SHSY-5Y).**


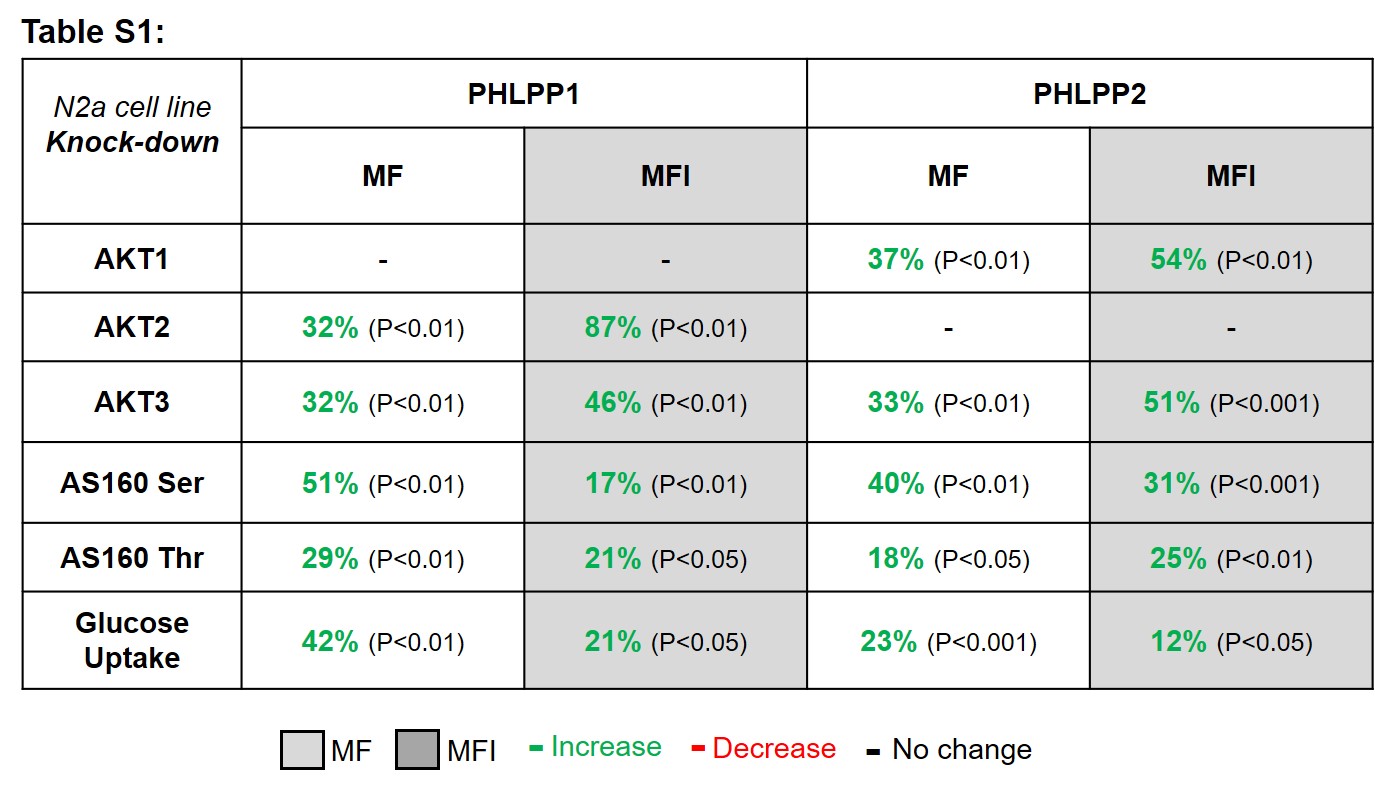


**Table S1: Effect of PHLPP1 or PHLPP2 silencing on Akt isoforms, AS160 and neuronal glucose uptake under insulin resistant condition in neuronal cells (N2A).**


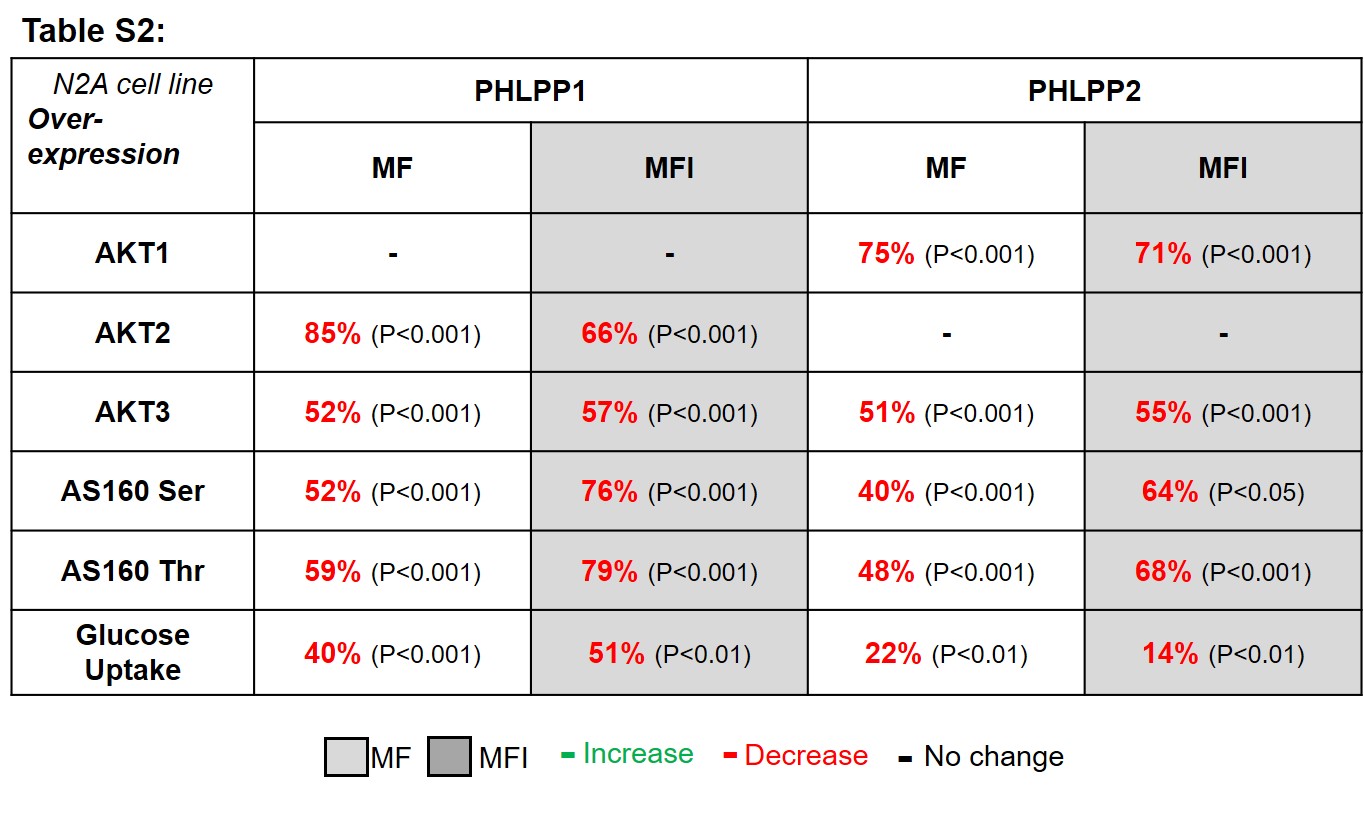


**Table S2: Effect of PHLPP1 or PHLPP2 over-expression on Akt isoforms, AS160 and neuronal glucose uptake under insulin resistant condition in neuronal cells (N2A).**


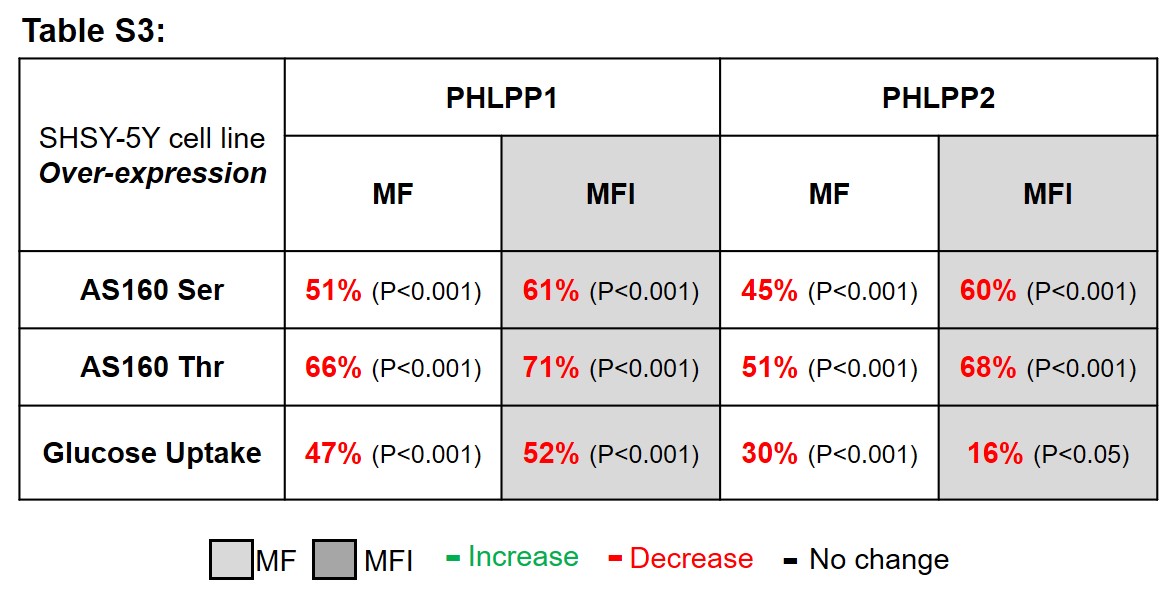


**Table S3: Effect of PHLPP1 or PHLPP2 over-expression on AS160 and neuronal glucose uptake under insulin resistant condition in neuronal cells (SHSY-5Y).**
